# Supplementary material for: Structural mechanisms for centrosomal recruitment and organization of the microtubule nucleator γ-TuRC
Source: Nat Commun. 2025 Mar 12;16:2453. doi: 10.1038/s41467-025-57729-2 (PMC11903878; doi:10.1038/s41467-025-57729-2)
Supplement: Supplementary file 1 — Supplementary Information [file 41467_2025_57729_MOESM1_ESM.pdf]

# Supplementary Information for

# Structural mechanisms for centrosomal recruitment and organization of the microtubule nucleator γ-TuRC

**Qi Gao[1,*], Florian W. Hofer[1,*], Sebastian Filbeck[1,*], Bram J.A. Vermeulen[1,*], Martin Würtz[1,2], Annett Neuner[1], Charlotte Kaplan[3], Maja Zezlina[1], Cornelia Sala[1], Hyesu Shin[1], Oliver J. Gruss[4], Elmar Schiebel[1,#] and Stefan Pfeffer[1,#]**

[1] Zentrum für Molekulare Biologie der Universität Heidelberg (ZMBH); Germany.

[2] European Molecular Biology Laboratory (EMBL); Heidelberg

[3] BioQuant, Universität Heidelberg; Germany.

[4] Institut für Genetik, Universität Bonn; Bonn, Germany.

* These authors contributed equally

[#] All correspondence should be addressed to Stefan Pfeffer (s.pfeffer@zmbh.uni-heidelberg.de) or Elmar Schiebel (e.schiebel@zmbh.uni-heidelberg.de)

# Supplementary Figures

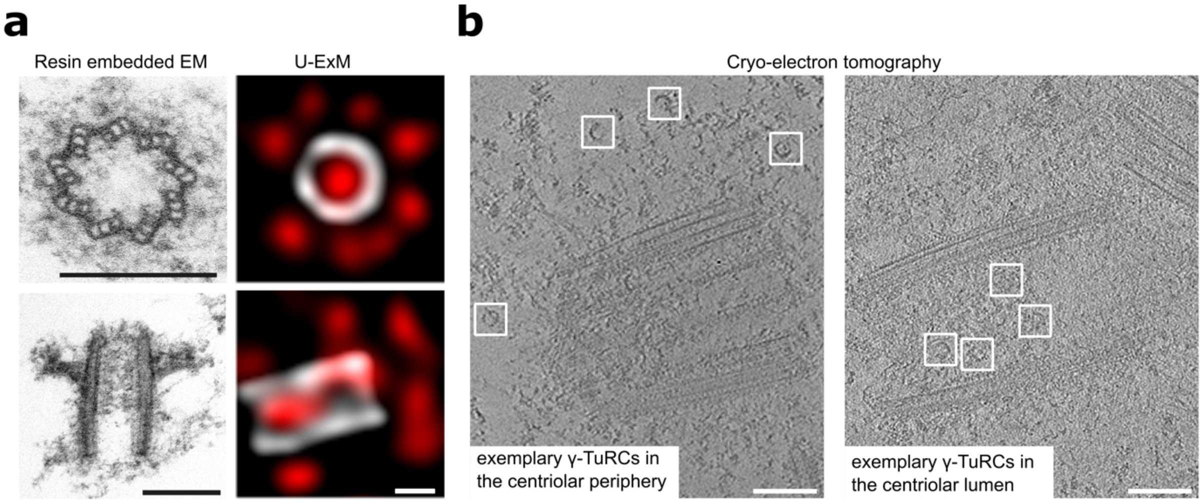


**Supplementary Fig. 1. Structural characterization of centrosomes purified from KE37 cells. a**) Representative EM (left) and U-ExM (right) images of isolated centrioles from KE37 cells. Before EM imaging, centrosomes were resin-embedded and stained with uranyl acetate. For U-ExM imaging, centrosomes were stained against γ-tubulin (red) and $\alpha$-tubulin (grey). N=3 biologically independent experiments. Scale bars: 200 nm (EM) and 100 nm (U-ExM). **b**) Z-slices through cryo-electron tomograms of isolated centrosomes (n=50). Exemplary pericentriolar (left) and lumenal (right) γ-TuRCs are highlighted. Scale bar: 100 nm.

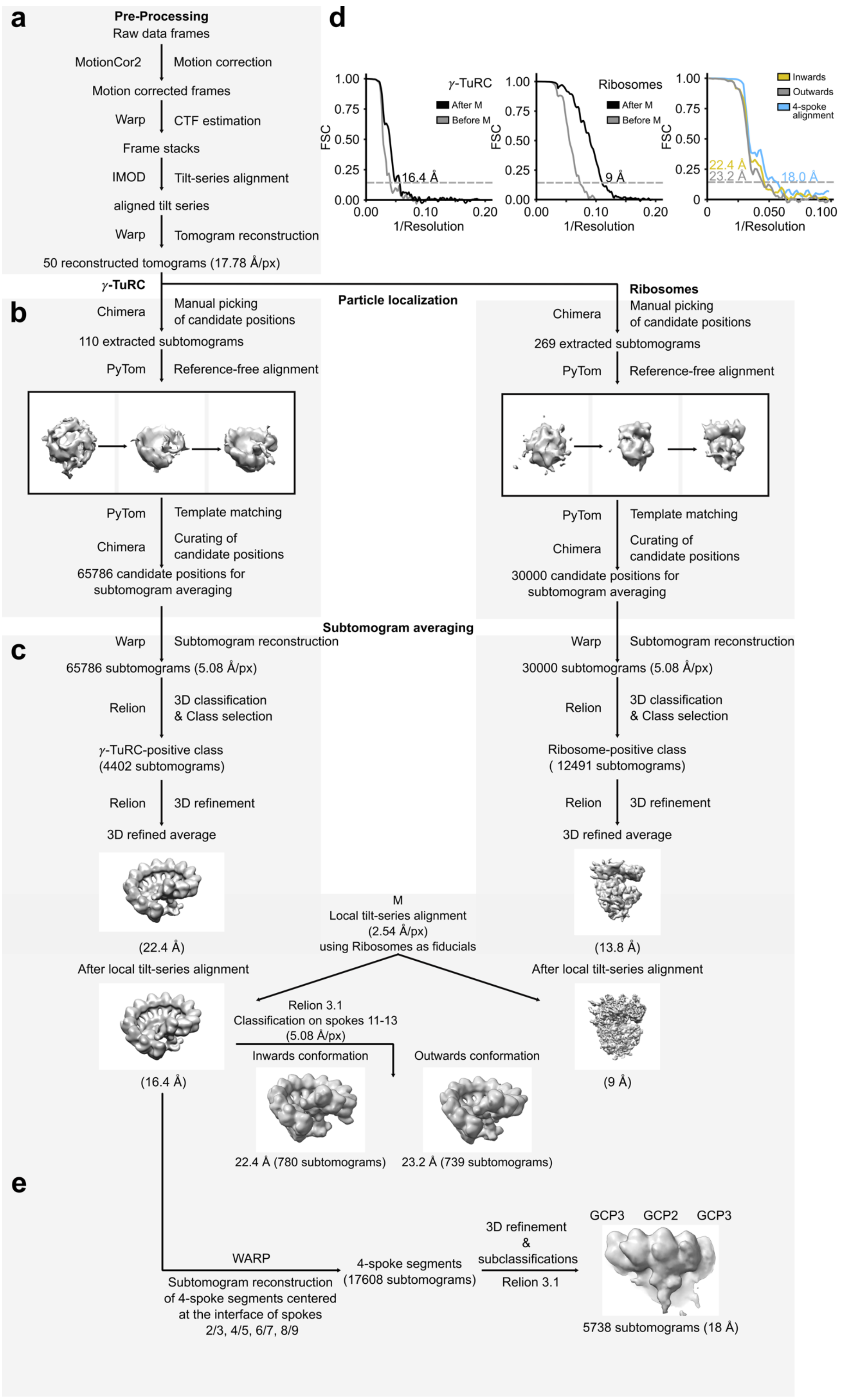
a
Pre-Processing
Raw data frames
MotionCor2
Motion correction
Motion corrected frames
Warp
CTF estimation
Frame stacks
IMOD
Tilt-series alignment
aligned tilt series
Warp
Tomogram reconstruction
50 reconstructed tomograms (17.78 Å/px)
d
1.00
0.75
0.50
0.25
0.00
FSC
γ-TuRC
After M
Before M
16.4 Å
0.00
0.10
0.20
1/Resolution
Ribosomes
After M
Before M
9 Å
Inwards
Outwards
4-spoke alignment
22.4 Å
23.2 Å
18.0 Å
0
0.050
0.100
γ-TuRC
Ribosomes
b
Particle localization
Chimera
Manual picking of candidate positions
110 extracted subtomograms
269 extracted subtomograms
PyTom
Reference-free alignment
PyTom
Template matching
Chimera
Curating of candidate positions
65786 candidate positions for subtomogram averaging
30000 candidate positions for subtomogram averaging
c
Subtomogram averaging
Warp
Subtomogram reconstruction
65786 subtomograms (5.08 Å/px)
30000 subtomograms (5.08 Å/px)
Relion
3D classification & Class selection
γ-TuRC-positive class (4402 subtomograms)
Ribosome-positive class ( 12491 subtomograms)
Relion
3D refinement
3D refined average
(22.4 Å)
(13.8 Å)
M
Local tilt-series alignment
(2.54 Å/px)
using Ribosomes as fiducials
After local tilt-series alignment
(16.4 Å)
(9 Å)
Relion 3.1
Classification on spokes 11-13
(5.08 Å/px)
Inwards conformation
Outwards conformation
22.4 Å (780 subtomograms)
23.2 Å (739 subtomograms)
e
WARP
Subtomogram reconstruction of 4-spoke segments centered at the interface of spokes 2/3, 4/5, 6/7, 8/9
4-spoke segments (17608 subtomograms)
3D refinement & subclassifications
Relion 3.1
GCP3
GCP2
GCP3
5738 subtomograms (18 Å)

**Supplementary Fig. 2. Processing scheme for purified centrosomes from KE37 cells. a**) Tilt series were pre-processed in MotionCor2 [1] and Warp [2]. Frame stacks were exported from Warp and aligned in IMOD [3,4] by gold-bead fiducial tracking. The resulting alignment parameters were used for tomogram reconstruction in Warp. **b**, **c**) Particle localization and subtomogram averaging of γ-TuRCs and ribosomes. A reference for template matching was obtained by reference-free alignment of manually selected candidate particles in PyTom [5]. Resulting cross-correlation peaks from template matching in PyTom were manually curated and used for subtomogram reconstruction in Warp. Subtomograms were subjected to 3D classification followed by 3D refinement in RELION 3.1 [6,7]. Tilt series were locally aligned in M using ribosomes as fiducial markers [8]. **d**) Mask-corrected FSC of independently refined half-set reconstructions for γ-TuRCs (left) and ribosomes (right) before and after local tilt-series alignment in M, as well as Inwards, Outwards and 4-spoke segment refinements in RELION 3.1. The FSC cut-off criterion of 0.143 has been marked in grey dashed lines. **e)** γ-TuSC-containing 4-spoked density segments were individually reconstructed for the symmetric half of the γ-TuRC. Following 3D auto-refinement in RELION 3.1, subtomograms were classified for the presence of CM1 modules. The retained particles were subjected to another round of 3D auto-refinement.

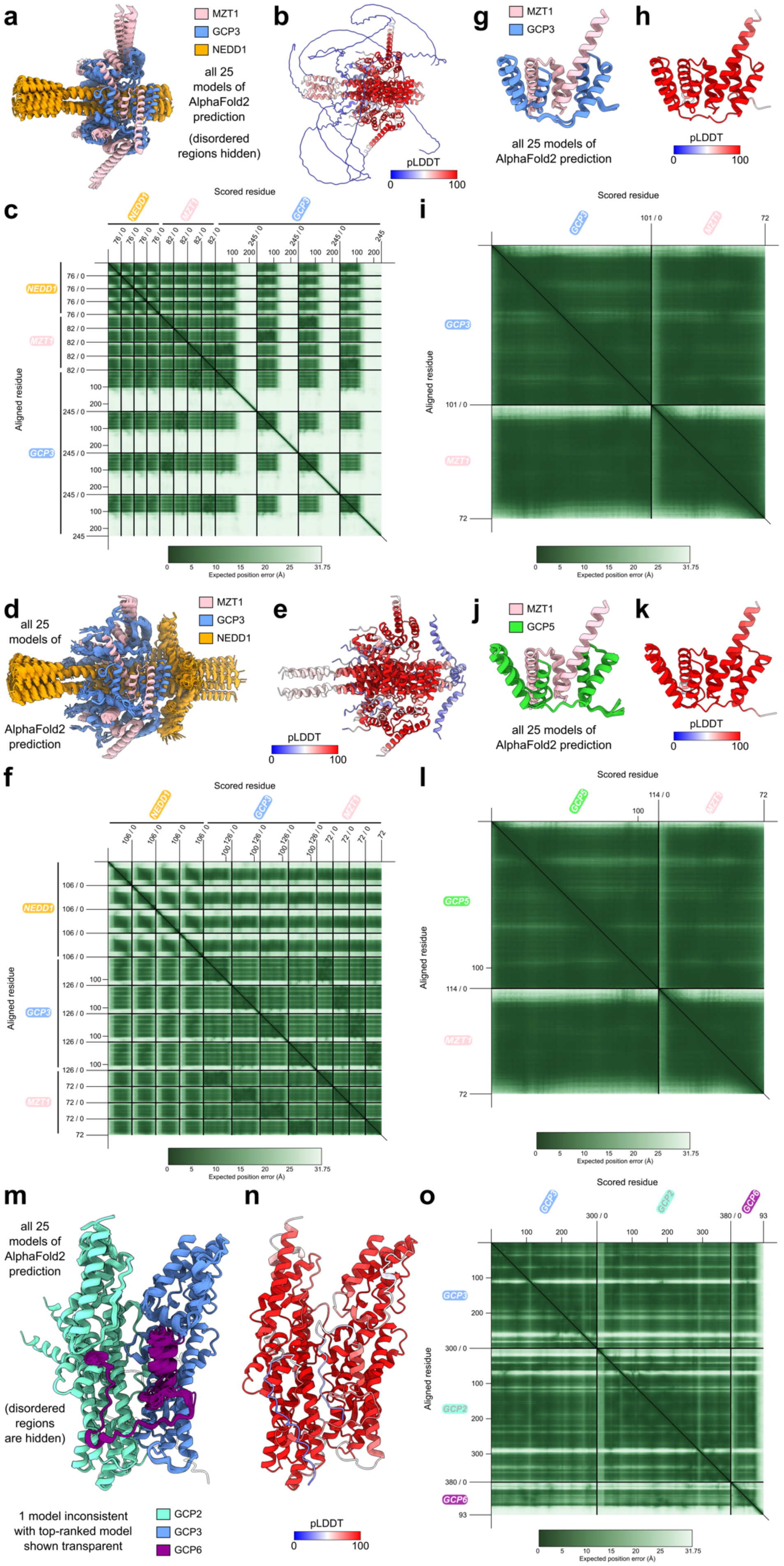

a
MZT1
GCP3
NEDD1
all 25 models of AlphaFold2 prediction
(disordered regions hidden)
b
pLDDT
0
100
g
MZT1
GCP3
all 25 models of AlphaFold2 prediction
h
pLDDT
0
100
c
Scored residue
NEDD1
MZT1
GCP3
Aligned residue
Expected position error (Å)
i
Scored residue
GCP3
MZT1
Aligned residue
Expected position error (Å)
d
all 25 models of AlphaFold2 prediction
MZT1
GCP3
NEDD1
e
pLDDT
0
100
j
MZT1
GCP5
all 25 models of AlphaFold2 prediction
k
pLDDT
0
100
f
Scored residue
NEDD1
GCP3
MZT1
Aligned residue
Expected position error (Å)
l
Scored residue
GCP5
MZT1
Aligned residue
Expected position error (Å)
m
all 25 models of AlphaFold2 prediction
(disordered regions are hidden)
1 model inconsistent with top-ranked model shown transparent
GCP2
GCP3
GCP6
n
pLDDT
0
100
o
Scored residue
GCP3
GCP2
GCP6
Aligned residue
Expected position error (Å)

**Supplementary Fig. 3: AlphaFold2 predictions of NEDD1 grapnel, N-GCP/MZT1 modules and N-GCP6 on GCP2 and GCP3. a-f)** Full ensemble of 25 models for the AlphaFold2 prediction of the human (**a**) and *X. laevis* (**d**) N-GCP3/MZT1/NEDD1 grapnel aligned on a single MZT1 chain of the top-ranked model (in panel A, disordered regions were hidden), top-ranked model including any disordered regions colored by pLDDT (predicted local distance difference test, **b** and **e**) and the associated PAE (predicted aligned error) matrix (**c** and **f**). **g-i)** Full ensemble of 25 models for the AlphaFold2 prediction of the *X. laevis* N-GCP3/MZT1 module aligned on the MZT1 chain of the top-ranked model (**g**), top-ranked model colored by pLDDT (**h**) and the associated PAE matrix (**i**). **j-l**) Full ensemble of 25 models for the AlphaFold2 prediction of the *X. laevis* N-GCP5/MZT1 module aligned on the MZT1 chain of the top-ranked model (**j**), top-ranked model colored by pLDDT (**k**) and the associated PAE matrix (**l**). **m-o**) Full ensemble of 25 models for the AlphaFold2 prediction of the interaction between N-GCP6 and spoke 1/2 in *X. laevis* aligned on the GCP3 chain of the top-ranked model (**m**, disordered regions were hidden; the 1 model inconsistent with the top-ranked model is shown transparent), top-ranked model colored by pLDDT (**n**) and the associated PAE matrix (**o**). Color schemes are indicated. Numbering in PAE matrices starts at the first residue included in the prediction, i.e., not necessarily at the N-terminus of the full-length protein, see also method section. PAE matrices were produced using the PAE Viewer webserver [9].

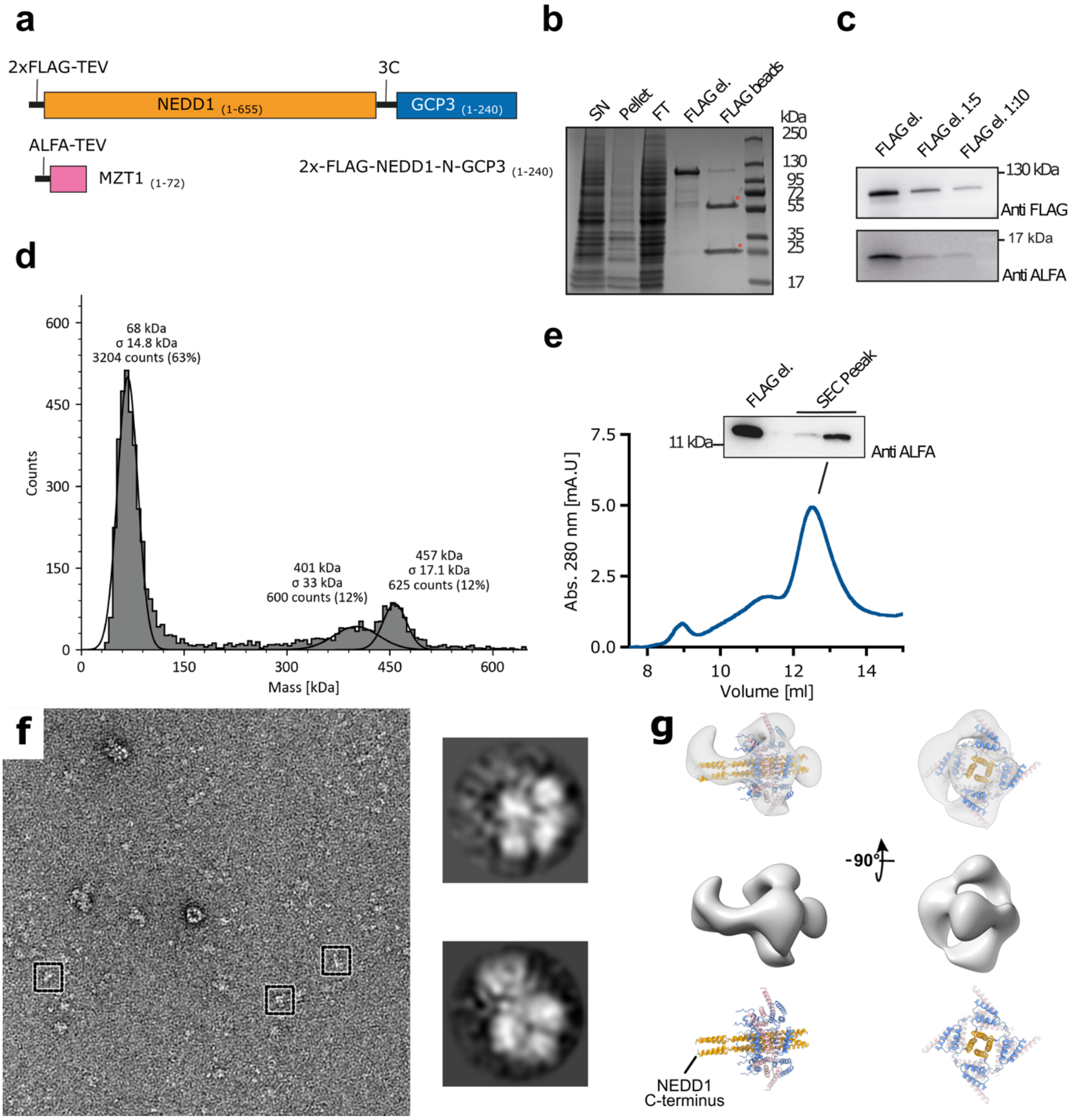


**Supplementary Fig. 4: NEDD1-N-GCP3/MZT1 complexes tetramerize and recapitulate the grapnel structure in negative stain EM. a)** Insect cell expression construct of *Xenopus laevis* NEDD1-N-GCP3(1-240) fusion construct, co-expressed with MZT1. **b)** SDS-PAGE analysis of FLAG-purified NEDD1-N-GCP3/MZT1 complex. Red asterisks indicate heavy and light chains from the anti-FLAG resin. **c)** Immunoblot analysis of the FLAG elution shown in panel b at different concentrations. Immunoblots were performed with anti-FLAG and anti-ALFA-tag antibodies. **d)** Mass photometry analysis of the FLAG elution of the NEDD1-N-GCP3/MZT1 complex. Mass distribution shows evidence of complex formation corresponding to a tetramer (460 kDa), despite a background of lower molecular weight species. The experiment was repeated with N=2 biological replicates. **e)** SEC chromatogram of the NEDD1-N-GCP3/MZT1 FLAG

purification, including immunoblot analysis of the elution and peak fractions against the ALFA-tag. The peak fractions were used for negative stain EM. **f)** Left: exemplary negative stain EM micrograph (n=200) of the NEDD1-N-GCP3/MZT1 peak fraction shown in panel e. Particle positions indicated by boxes. Right: representative 2D class averages. **g)** 3D reconstruction of NEDD1-N-GCP3/MZT1 from *Xenopus laevis* generated in an entirely data-driven and reference-free manner by ab-initio model generation in RELION 3.1. The density was low-pass filtered to 35 Å resolution and the AlphaFold2 prediction of the *Xenopus laevis* NEDD1-N-GCP3/MZT1 complex superposed. Source data are provided as a Source Data file.

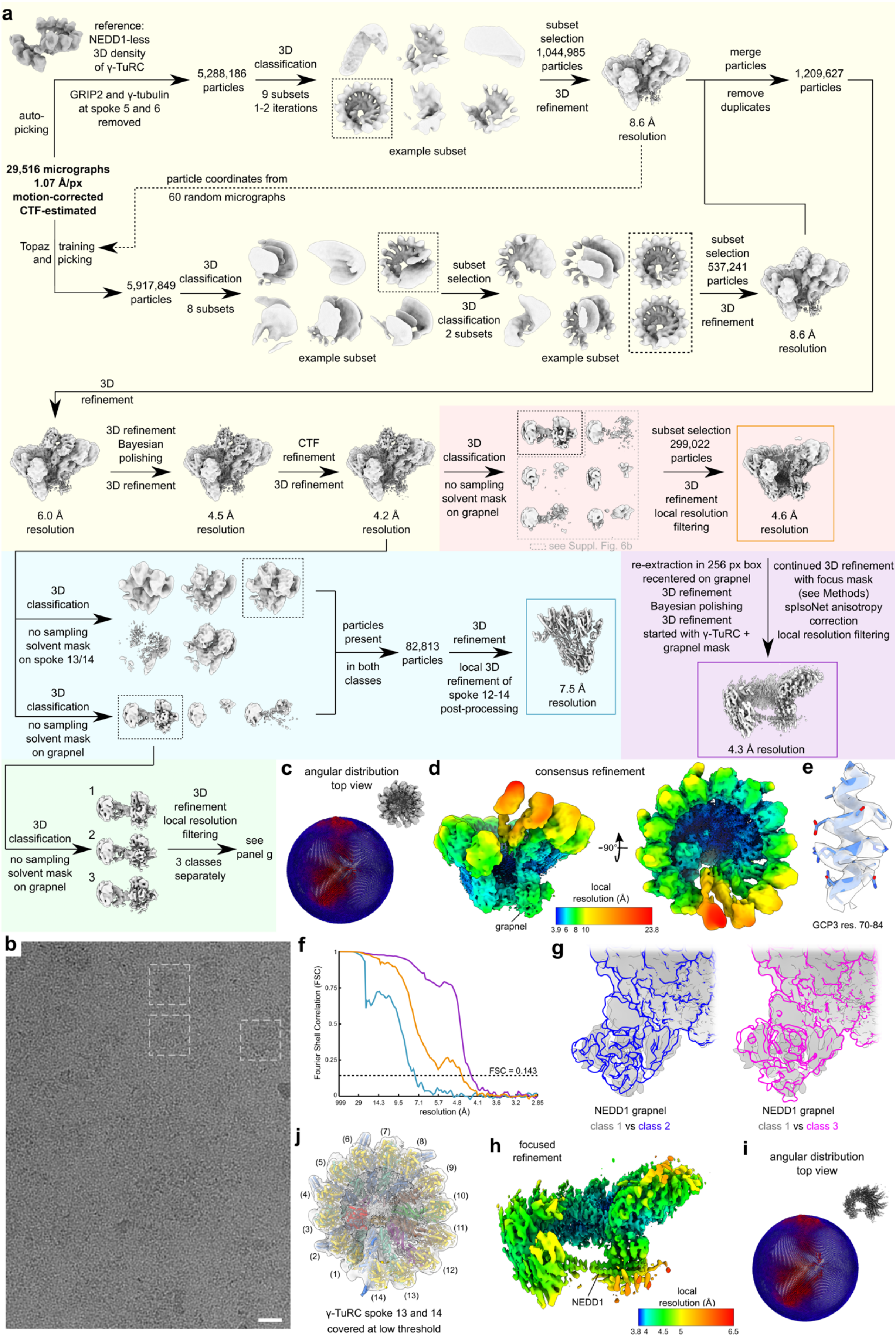
a
reference: NEDD1-less 3D density of γ-TuRC
GRIP2 and γ-tubulin at spoke 5 and 6 removed
auto-picking
5,288,186 particles
3D classification
9 subsets 1-2 iterations
example subset
subset selection 1,044,985 particles
3D refinement
8.6 Å resolution
merge particles
remove duplicates
1,209,627 particles
29,516 micrographs
1.07 Å/px
motion-corrected
CTF-estimated
particle coordinates from 60 random micrographs
Topaz training and picking
5,917,849 particles
3D classification
8 subsets
example subset
subset selection
3D classification 2 subsets
example subset
subset selection 537,241 particles
3D refinement
8.6 Å resolution
3D refinement
6.0 Å resolution
3D refinement Bayesian polishing
3D refinement
4.5 Å resolution
CTF refinement
3D refinement
4.2 Å resolution
3D classification
no sampling solvent mask on grapnel
see Suppl. Fig. 6b
subset selection 299,022 particles
3D refinement local resolution filtering
4.6 Å resolution
3D classification
no sampling solvent mask on spoke 13/14
3D classification
no sampling solvent mask on grapnel
particles present
in both classes
82,813 particles
3D refinement
local 3D refinement of spoke 12-14 post-processing
7.5 Å resolution
re-extraction in 256 px box recentered on grapnel
3D refinement
Bayesian polishing
3D refinement
started with γ-TuRC + grapnel mask
continued 3D refinement with focus mask (see Methods)
spIsoNet anisotropy correction
local resolution filtering
4.3 Å resolution
3D classification
no sampling solvent mask on grapnel
1
2
3
3D refinement local resolution filtering
3 classes separately
see panel g
c
angular distribution top view
d
consensus refinement
-90°
local resolution (Å)
3.9 6 8 10 23.8
grapnel
e
GCP3 res. 70-84
b
f
Fourier Shell Correlation (FSC)
FSC = 0.143
resolution (Å)
999 29 14.3 9.5 7.1 5.7 4.8 4.1 3.6 3.2 2.85
g
NEDD1 grapnel
class 1 vs class 2
NEDD1 grapnel
class 1 vs class 3
j
(1) (2) (3) (4) (5) (6) (7) (8) (9) (10) (11) (12) (13) (14)
γ-TuRC spoke 13 and 14 covered at low threshold
h
focused refinement
NEDD1
local resolution (Å)
3.8 4 4.5 5 6.5
i
angular distribution top view

**Supplementary Fig. 5. Cryo-EM SPA data processing and analysis of the *X. laevis* γ-TuRC with NEDD1. a**) Detailed data processing scheme. After pre-processing, selection, sorting and alignment of high-quality true-positive γ-TuRC particles (yellow background), particles were classified and refined to yield a consensus reconstruction of the γ-TuRC with NEDD1 grapnel (red background), classified to yield a well-defined reconstruction of the N-GCP5/MZT1 module on spoke 14 on a particle set with stoichiometric NEDD1 grapnel (blue background), subjected to further focused refinement to increase resolution for NEDD1 (purple background) or classified to visualize the flexibility of the NEDD1 grapnel with respect to the γ-TuRC core (green background). **b**) Cut-out from an exemplary micrograph (n=29516). Example particles are indicated by dashed boxes. Scale bar: 25 nm. **c**) Angular distribution of particles used in the reconstruction after consensus refinement of the γ-TuRC with NEDD1 (orange box in panel a), viewed from the top, as indicated by the small reconstruction (grey, top right). **d**) Reconstruction after consensus refinement of the γ-TuRC with NEDD1 colored according to local resolution. **e**) Exemplary helix (GCP3 residue 70-84 in the lumenal bridge) in the most highly-resolved region of the consensus reconstruction displaying side chain resolution, fitted with the corresponding model after MDFF. **f**) Fourier Shell Correlation (FSC) curves for reconstructions outlined by blue, orange and purple boxes in panel a. FSC threshold at 0.143 is indicated. **g**) The NEDD1 grapnel displays conformational flexibility with respect to the γ-TuRC core. 3D-refined classes filtered to local resolution resulting from classification (green background in panel a) are overlaid and colored as indicated. Classes were filtered to local resolution in RELION 3.0. **h**) Reconstruction after focused refinement (purple box in panel a), colored according to local resolution. View as in panel d. **i**) Angular distribution of particles used in the reconstruction after focused refinement, viewed from the top, as indicated by the small reconstruction (grey, top right). **j**) Consensus reconstruction of the γ-TuRC with NEDD1 grapnel shown at low density threshold level, showing coverage of γ-TuRC spokes 13 and 14. Spoke numbering is indicated.

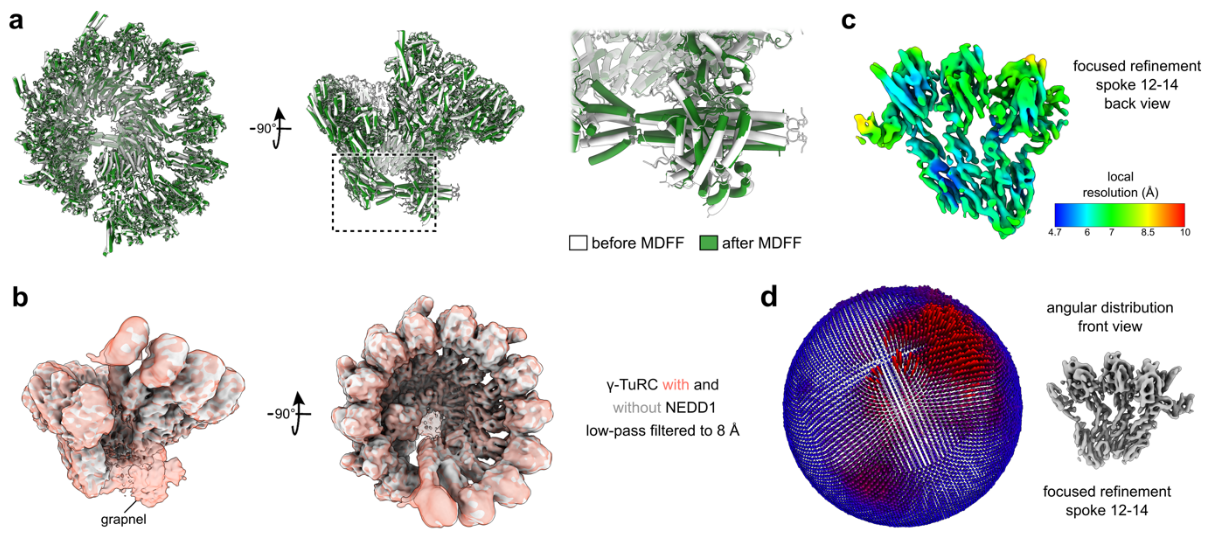


**Supplementary Fig. 6. Conformation of the *X. laevis* γ-TuRC with NEDD1 and data processing for spoke 12 to 14 of the γ-TuRC. a**) Models before (white) and after MDFF (green) in the consensus reconstruction of the *X. laevis* γ-TuRC with NEDD1 are superimposed [10], showing minimal changes apart from the four $NEDD1^{651-670}$ helices. **b**) The conformation of the γ-TuRC core is indistinguishable in the presence (red) and absence (grey) of NEDD1. Local-resolution filtered consensus reconstructions were low-pass filtered to 8 Å to aid visualization (local-resolution filtering and low-pass filtering performed in RELION 3.0). **c**) Reconstruction after refinement focused on spoke 12 to 14 of the γ-TuRC (blue box in Supplementary Fig. 5a), colored according to local resolution. **d**) Angular distribution of particles used in the reconstruction after refinement focused on spoke 12 to 14 of the γ-TuRC, viewed from the front, as indicated by the small reconstruction (grey, right).

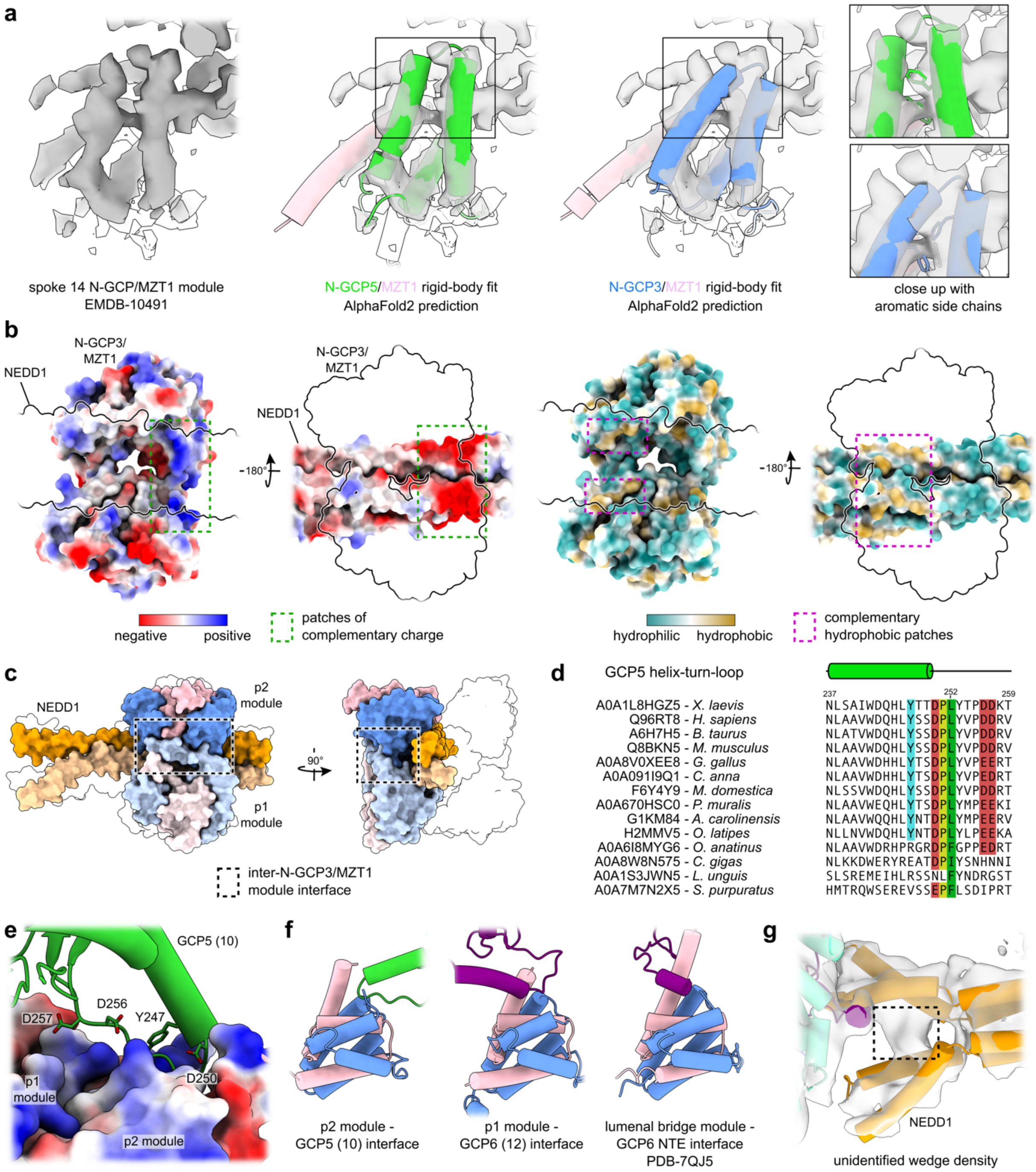


**Supplementary Fig. 7. Analysis of N-GCP/MZT1 modules and their protein-protein interfaces in the *X. laevis* γ-TuRC with NEDD1. a)** Rigid-body fit of AlphaFold2 predictions for *X. laevis* N-GCP5/MZT1 (center-left panel) and N-GCP3/MZT1 (center-right panel) modules into the density for the N-GCP/MZT1 module on spoke 14 in the native *X. laevis* γ-TuRC (EMD 10491, left) [11]. Close-ups on the right display the area of largest deviation between the N-GCP/MZT1 modules, additionally highlighting aromatic sidechains that match corresponding density for the N-GCP5/MZT1 but not N-GCP3/MZT1 module. Corresponding cross correlation values and fit of cryo-EM and X-ray crystallography-derived N-GCP/MZT1 atomic models can

be found in Supplementary Table 3. Details on the N-GCP/MZT1 AlphaFold2 predictions are shown in Supplementary Fig. 3g-l. Coloring as indicated. **b)** Complementary electrostatic (left) and hydrophobic (right) patches that make up the interface between NEDD1 and N-GCP3/MZT1 in the grapnel, indicated by green and purple dashed boxes, respectively. For surfaces colored by electrostatics or hydrophobicity, outlines indicate the position of the corresponding binding patches. **c)** Surface representation of atomic models of the p1 and p2 N-GCP3/MZT1 modules in the NEDD1 grapnel, highlighting their interface with a dashed box. Coloring as in Fig. 2a, with pastel color variations for the p1 module. Outlines indicate the position of the remaining two N-GCP3/MZT1 modules and associated NEDD1 protomers. **d)** Sequence conservation of the GCP5 helix-turn-loop motif involved in NEDD1 grapnel binding among metazoans. UniProt IDs are provided. Residues shown in panel e or Fig. 2c are colored. Red, yellow, green and blue coloring indicates acidic, proline, large hydrophobic and polar aromatic residues, respectively. Secondary structure is indicated schematically. **e)** Electrostatic surface of the p1 and p2 N-GCP3/MZT1 modules at the interface with the helix-turn-loop motif of GCP5 at spoke 10. Acidic and aromatic residues in GCP5 that interact with the basic surface of the N-GCP3/MZT1 modules are shown; residue numbering is indicated. **f)** Comparison of N-GCP3/MZT1 interfaces involved in binding GCP5 at spoke 10 (left) as well as GCP6 at spoke 12 (middle) in the grapnel and the N-terminal extension (NTE) of GCP6 in the lumenal bridge (PDB 7QJ5) [12]. **g)** Close-up of the cryo-EM reconstruction of the NEDD1-containing γ-TuRC with the fit atomic model, highlighting the unidentified wedge density between the two pairs of coiled coils formed by $\text{NEDD1}^{651\text{-}670}$. Coloring in this figure as in Fig. 2a, unless otherwise indicated. The atomic model based on the consensus refinement of the NEDD1-containing γ-TuRC is shown in this figure, unless mentioned otherwise.

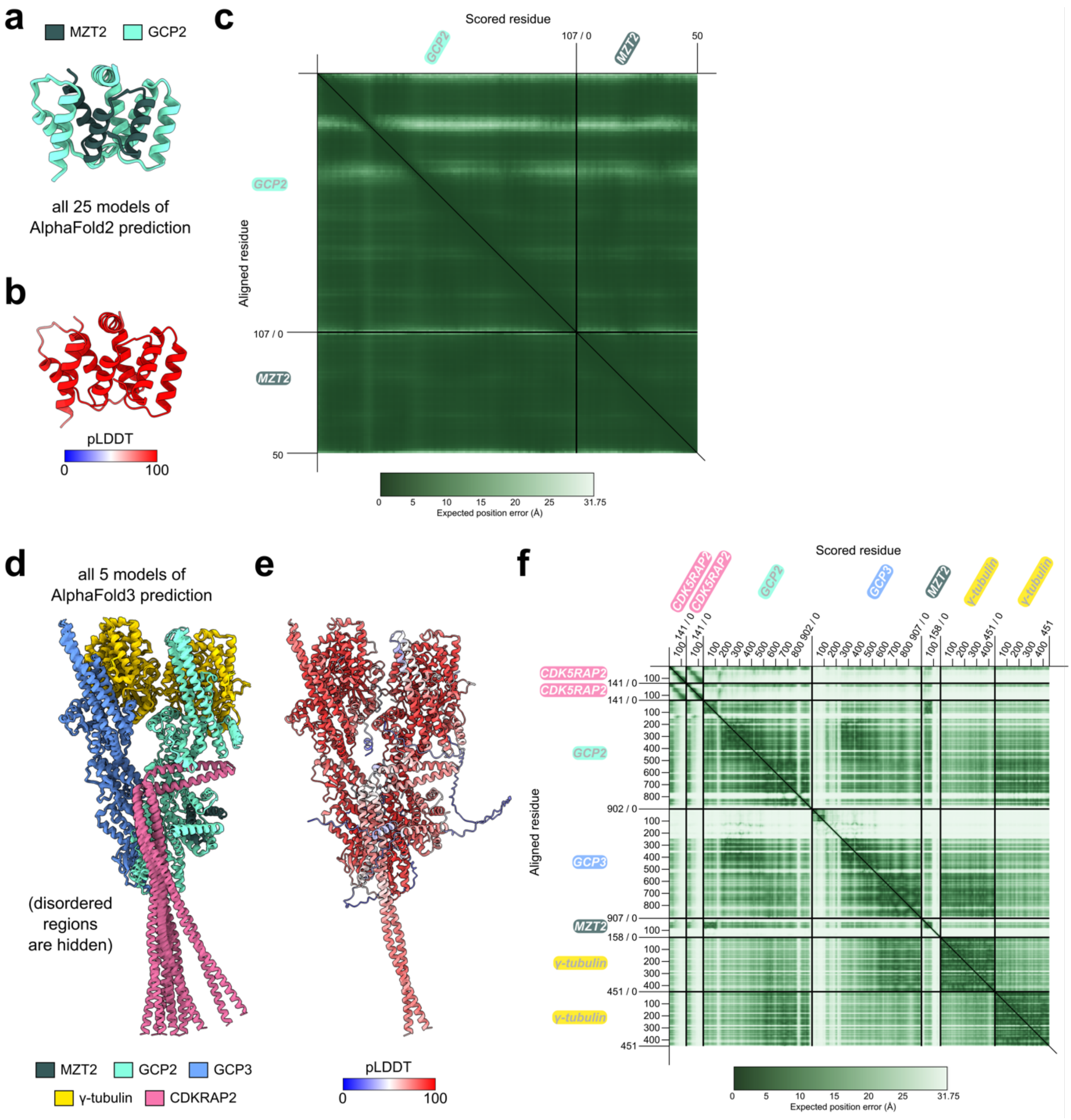


**Supplementary Fig. 8. AlphaFold predictions of the N-GCP2/MZT2 module in isolation and in complex with CDK5RAP2-γ-TuSC. a-c)** Full ensemble of 25 models for the AlphaFold2 prediction of the *X. laevis* N-GCP2/MZT2B module aligned on a single MZT2B chain of the top-ranked model (a) as well as the top-ranked model colored by pLDDT (predicted local distance difference test, b) and the associated PAE (predicted aligned error) matrix (c). **d-f)** Full ensemble of 5 models for the AlphaFold3 prediction of human GCP2, GCP3 and two copies of γ-tubulin in complex with MZT2 and a fragment of dimeric CDK5RAP2, aligned on the GCP2 chain of the top-ranked model (d, disordered regions were hidden), top-ranked model including disordered regions colored by pLDDT (e) and the associated PAE matrix (f). Color schemes are indicated. Numbering in PAE matrices starts at the first residue included in the

prediction, i.e., not necessarily at the N-terminus of the full-length protein, see also method section. PAE matrices were produced using the PAE Viewer webserver [9].

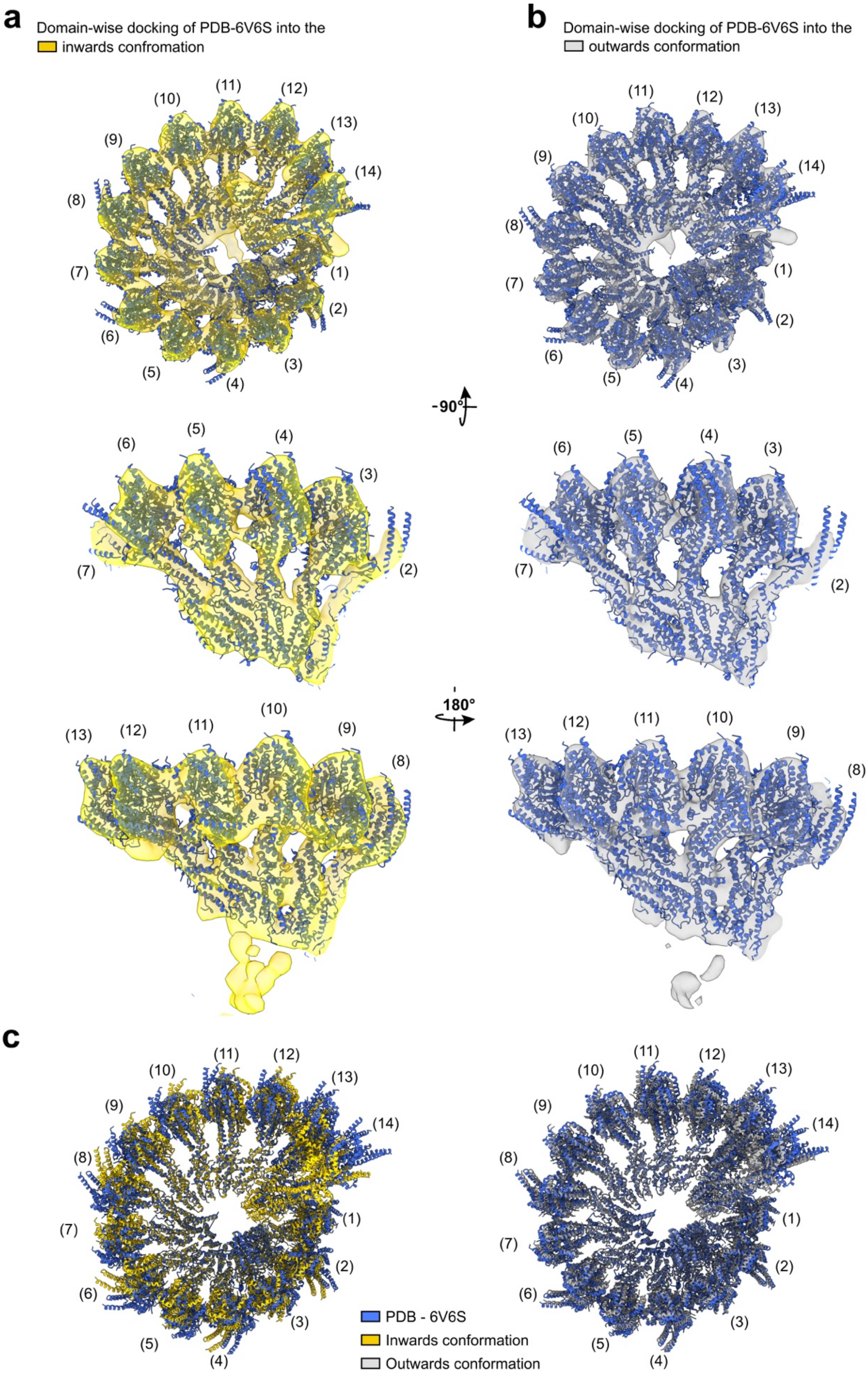
a
Domain-wise docking of PDB-6V6S into the
inwards confromation
b
Domain-wise docking of PDB-6V6S into the
outwards conformation
(1)
(2)
(3)
(4)
(5)
(6)
(7)
(8)
(9)
(10)
(11)
(12)
(13)
(14)
-90°
180°
c
PDB - 6V6S
Inwards conformation
Outwards conformation

**Supplementary Fig. 9. Domain-wise rigid body docking generates models for the inwards and outwards conformations of pericentriolar γ-TuRCs in centrosomes isolated from KE37 cells. a, b**) The atomic model of the isolated human γ-TuRC (PDB 6V6S) [13] was split into separate rigid bodies and docked into the inwards (a) and outwards (b) conformations of the pericentriolar γ-TuRC in purified KE37 centrosomes. See methods for details. **c**) The models for the inwards (left) and outwards (right) conformations were superposed to PDB 6V6S [13] based on the GRIP1 domains.

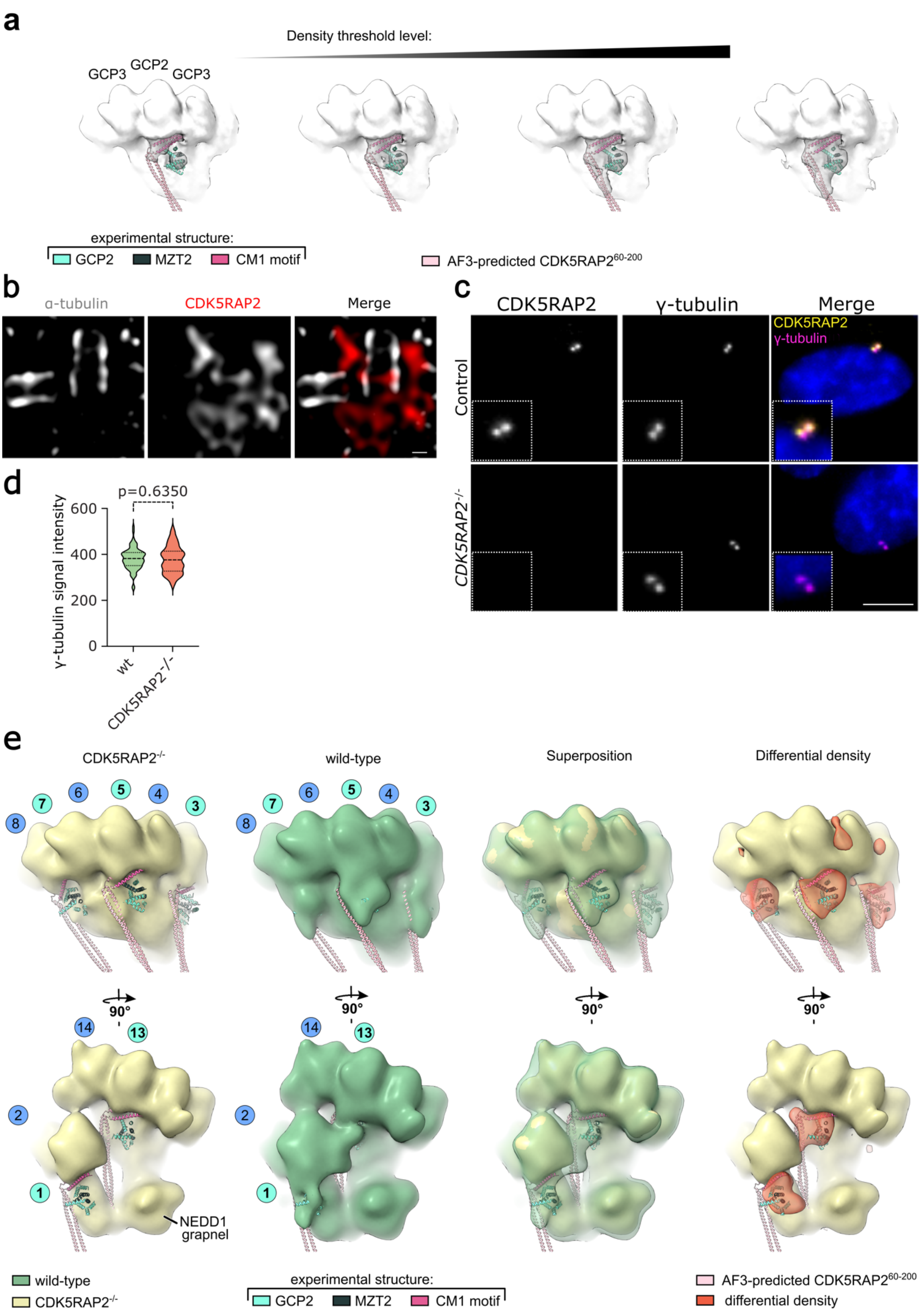


**Supplementary Fig. 10. CDK5RAP2-derived CM1 modules decorate PCM-localized γ-TuRCs in purified centrosomes. a)** Cryo-EM density of GCP2/3 units

(white) with additional CM1 module-derived density on the outside of GCP2 (grey) shown at different threshold levels. The experimental model (PDB 6V6S [13]) was fitted based on spokes 13 & 14 of human γ-TuRC and superposed with an AF3 prediction of CDK5RAP2$^{60-200}$ dimer together with a γ-TuSC and MZT2A (Supplementary Fig. 8d-f). **b)** U-ExM images of centrioles stained against CDK5RAP2 (red) and α-tubulin (grey), showing the localization of CDK5RAP2 in the PCM. N=3 biologically independent experiments. Scale bar: 200 nm. **c**) Immunofluorescence images of wild-type control and *CDK5RAP2*$^{-/-}$ cells stained against CDK5RAP2 (yellow), γ-tubulin (magenta), and DAPI (blue). Scale bar: 5 μm. **d**) Quantification of γ-tubulin fluorescence signal intensity in wild-type control and *CDK5RAP2*$^{-/-}$ cells from panel c plotted as mean ± SD, from N=3 independent experimental repeats. n > 60 cells per cell line for each experiment. Statistical analysis was performed using unpaired two-tailed t test. **e**) Low-pass-filtered (30 Å, relion_image_handler) and histogram-scaled (ChimeraX [14-16]) cryo-EM densities of pericentriolar γ-TuRCs from wild-type (green) and *CDK5RAP2*$^{-/-}$ RPE1 cells (gold) were superposed by FitMap in ChimeraX (correlation metric) and subtracted from each other. Positive density difference localized to CM1 module positions on all GCP2 subunits (red). The atomic model for the GCP2-associated CM1 module (CM1/MZT2A/N-GCP2) was fitted based on γ-tubulins and GCP2/3 subunits from human γ-TuRC spokes 13 & 14 (PDB: 6V6S [13]) and the AF3 prediction of CDK5RAP2$^{60-200}$ was matched on top of the experimental CM1 model in ChimeraX. Source data are provided as a Source Data file.

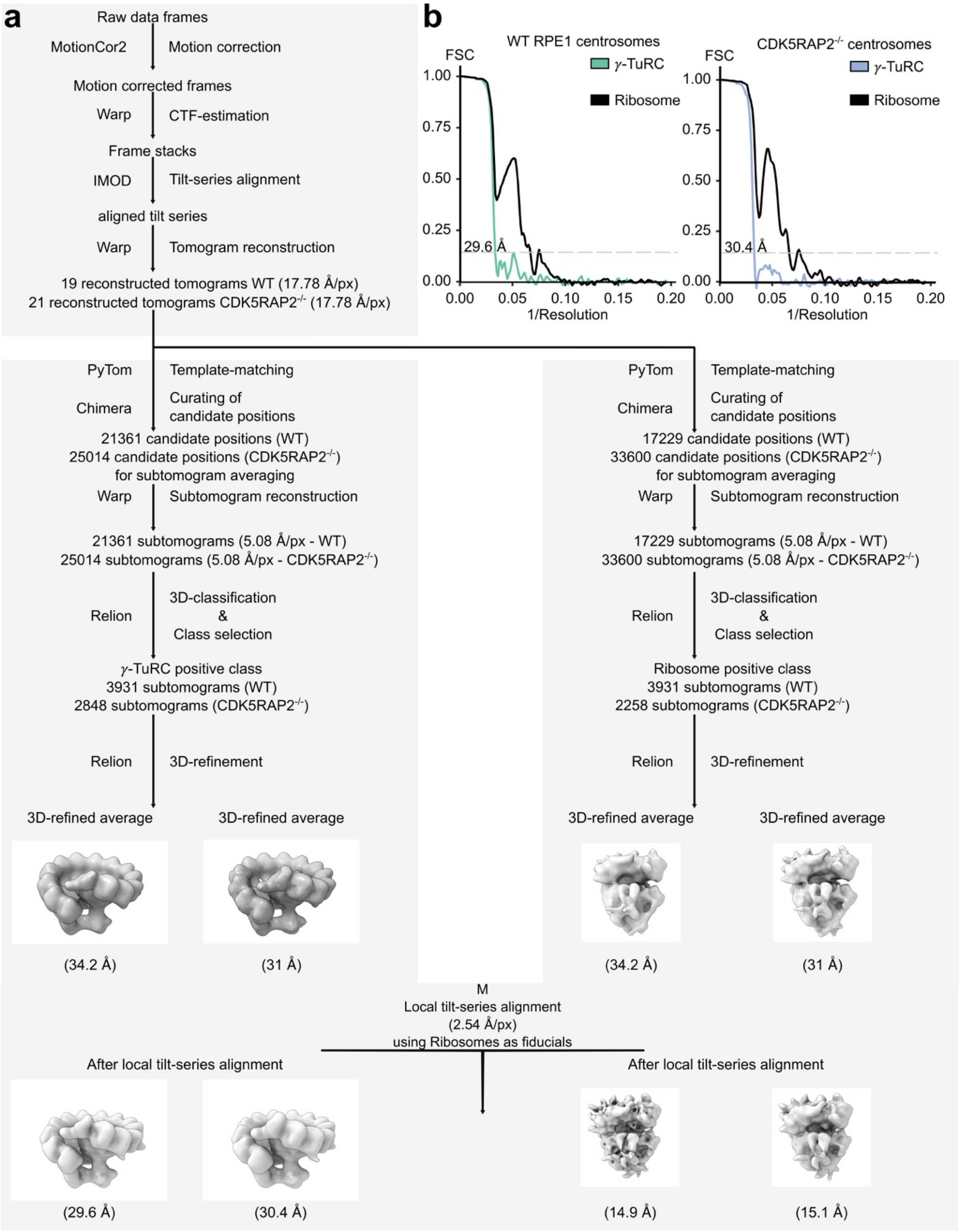


**Supplementary Fig. 11. Processing scheme for purified centrosomes from *CDK5RAP2*$^{-/-}$ and wild-type RPE1 cells.** **a**) Tilt-series pre-processing, particle localization and subtomogram averaging of γ-TuRCs and ribosomes were performed as in Supplementary Fig. 2. **b**) Mask-corrected FSC of independently refined half-set reconstructions for γ-TuRCs and ribosomes from wild-type (left) and *CDK5RAP2*$^{-/-}$

RPE1 cells (right) after local tilt-series alignment in M. The FSC cut-off criterion of 0.143 has been marked in grey.

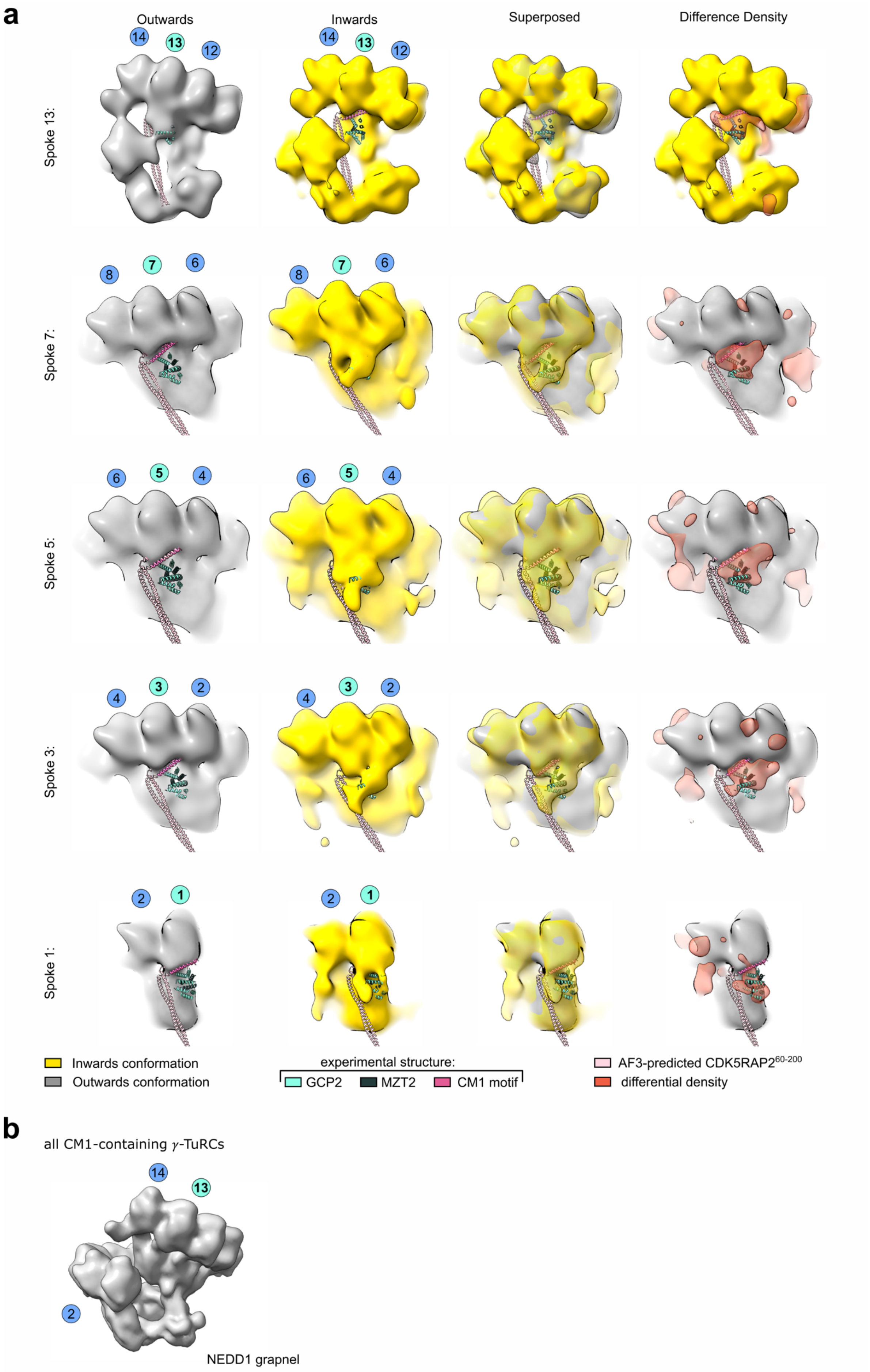

a
Outwards
Inwards
Superposed
Difference Density
Spoke 13:
14
13
12
Spoke 7:
8
7
6
Spoke 5:
6
5
4
Spoke 3:
4
3
2
Spoke 1:
2
1
Inwards conformation
Outwards conformation
experimental structure:
GCP2
MZT2
CM1 motif
AF3-predicted CDK5RAP2$^{60-200}$
differential density
b
all CM1-containing γ-TuRCs
14
13
2
NEDD1 grapnel

**Supplementary Fig. 12: Conformation-dependent pattern of CM1 module binding. a)** Low-pass-filtered (30 Å, relion_image_handler) and histogram-scaled (ChimeraX [14-16]) cryo-EM densities of the inwards (yellow) and outwards (grey) conformations were superposed by FitMap in ChimeraX (correlation metric) [14-16], and subtracted from each other. Positive density difference (red) localized to CM1 module positions on each GCP2 subunit is shown. Atomic models of the human GCP2-associated CM1 module (PDB: 6V6S [13]) and AF3 prediction extending CDK5RAP2 from residues 60-200 are superposed. Spoke numbers are indicated. Coloring as indicated. **b)** Cryo-EM reconstruction of all γ-TuRCs containing at least one CM1 module, as identified by subtomogram classification.

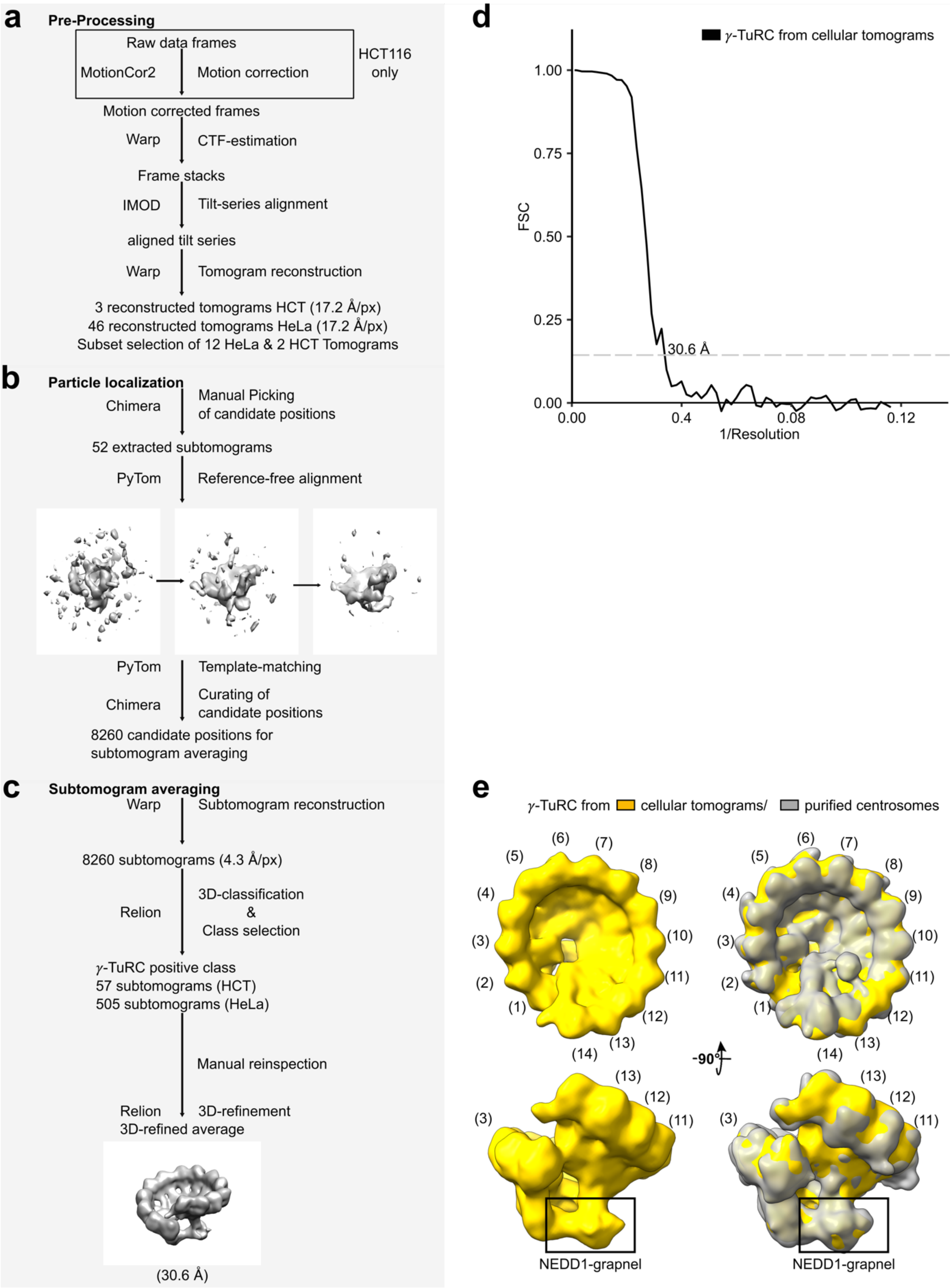


**Supplementary Fig. 13. Processing scheme for γ-TuRCs in cellular cryo-electron tomography. a, b**) Pre-processing and particle localization for tilt-series acquired on vitreous sections of HeLa [17] and HCT116 cells. Pre-processing of frames was done in MotionCor2 [1] (HCT116 only) and Warp [2]. Frame stacks were exported from Warp and

aligned in IMOD by patch tracking [3]. The resulting alignment parameters were used for tomogram reconstruction in Warp. 14 tomograms of sufficient quality were retained for further processing. Particle localization was carried out as for purified centrosomes (Supplementary Fig. 2b). **c**) Subtomogram averaging of γ-TuRCs was carried out as for purified centrosomes (Supplementary Fig. 2c). No improvement was observed after local tilt-series alignment in M. **d**) Mask-corrected FSC of independently refined half-set reconstructions. The FSC cut-off of 0.143 is indicated. **e**) Cryo-EM reconstructions of γ-TuRCs from purified centrosomes (grey) and vitreous cellular sections (yellow) were superposed.

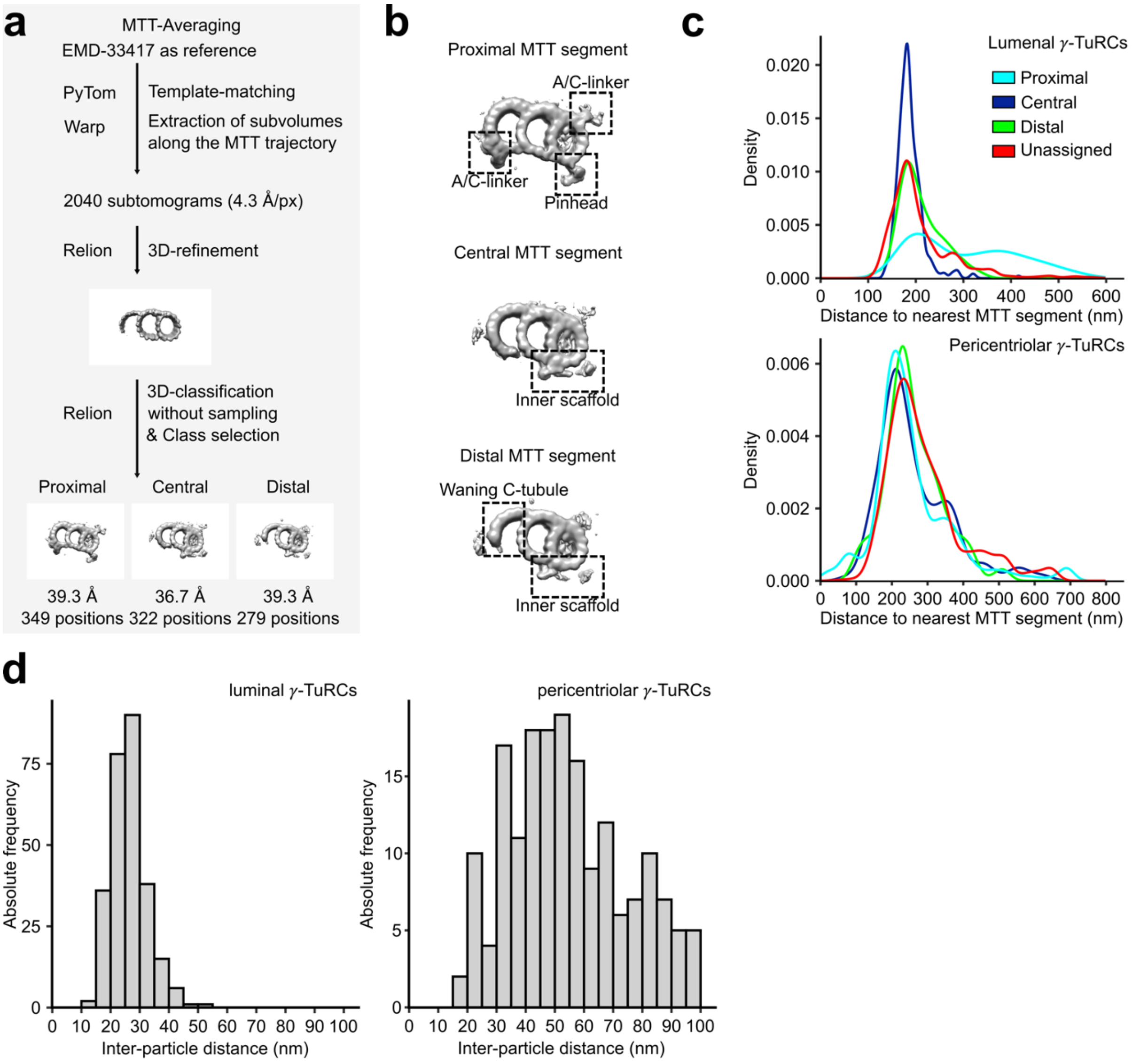


**Supplementary Fig. 14. Lumenal γ-TuRCs are densely packed in the central region of centrioles. a**) Processing scheme for MTTs in cellular tomograms of human centrioles. EMD 33417 [17] was used as a reference for template-matching. Subtomograms were reconstructed along the trajectories of maximal cross-correlation at approximately 8 nm spacing. Subtomograms were refined in RELION 3.1 and classified without sampling [6,7]. **b**) The 3D classes were assigned to different topological segments along the centriole axis based on distinct features. Proximal density segments: presence of A/C-linker and pinhead densities; central density segments: presence of inner-scaffold density on the lumenal side of the MTT; distal density segments: reduced number of protofilaments in the MTT C-tubule and inner-scaffold density on the lumenal side of the MTT. **c**) Distances between γ-TuRCs and the nearest MTT segment (n=15 centrioles distributed over 12 tomograms). Centriole lumenal γ-TuRCs are preferentially localized near central MTT segments. Pericentriolar γ-TuRCs are randomly distributed along the centriole axis. **d**) Distance

between γ-TuRCs and their closest neighbors (n=15 centrioles distributed over 12 tomograms). Lumenal γ-TuRCs (left) are densely packed with a center-to-center distance between 17.4 and 38.3 nm for 90% of particles, approximating the γ-TuRC diameter. Pericentriolar γ-TuRCs are spaced further apart with a center-to-center distance between 24.8 and 90.6 nm for 90% of particles. Source data are provided as a Source Data file.

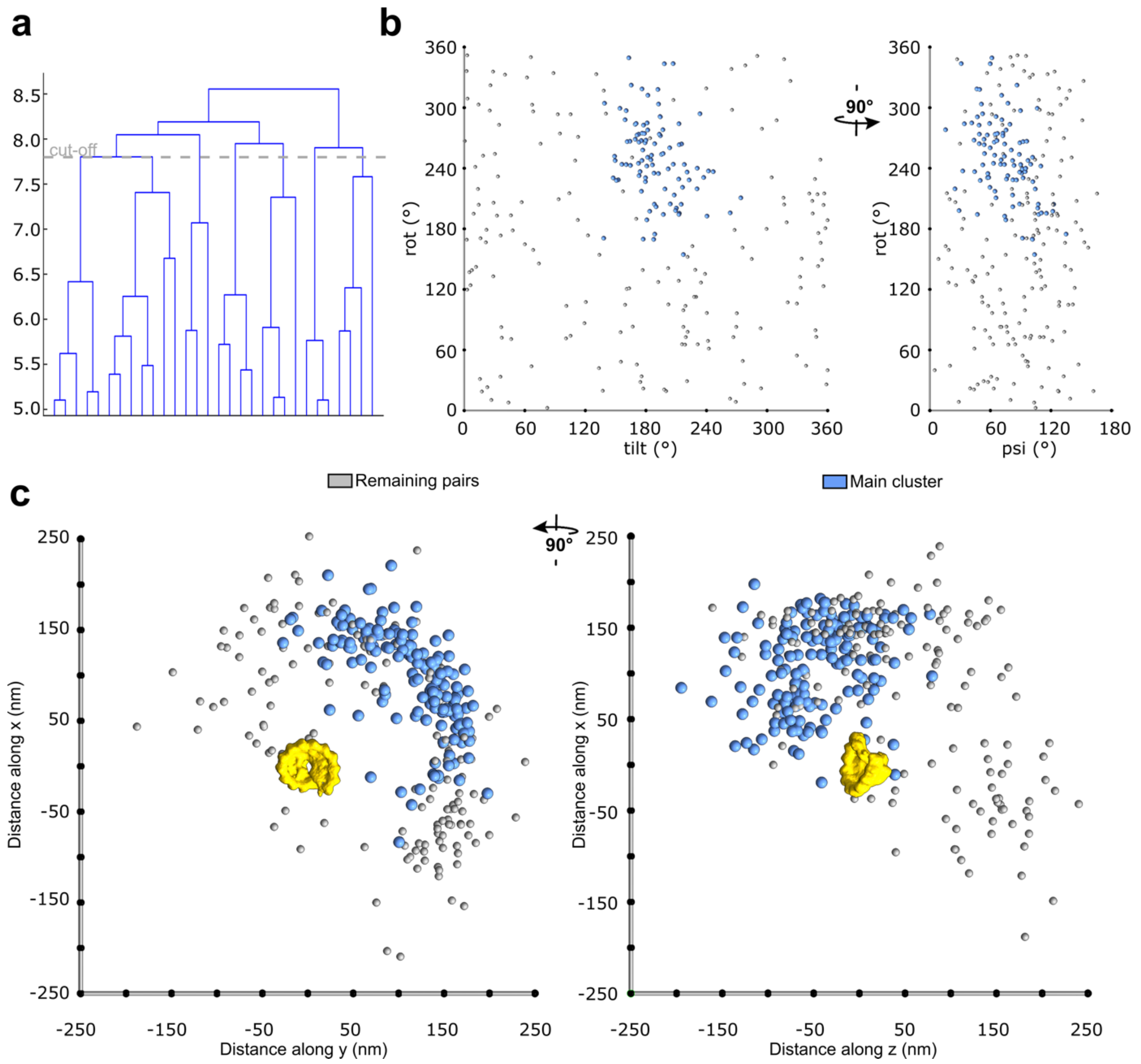


**Supplementary Fig. 15. γ-TuRCs adopt a defined spatial arrangement with respect to the inner centriolar wall.** The relative spatial arrangement of γ-TuRCs and the nearest MTT segments was analyzed by clustering of γ-TuRC-MTT pair configurations in coordinate and orientational space. One main cluster representing 42% of pair configurations was observed (blue). The remaining pair configurations are depicted in grey. **a)** Dendrogram reflecting the clustering of the pair configurations. The cut-off value of 7.9 is indicated. **b)** Scatter plot of Euler angles (tilt, rot, psi) representing the relative orientation between 232 γ-TuRCs and their nearest MTT-segments of n=15 centrioles, distributed over 12 tomograms, which were used for the final analysis. γ-TuRC-MTT pairs for which the MTT was incorrectly oriented during 3D auto-refinement were excluded from the analysis **c**) 3D coordinates of nearest MTT segment mapped into the coordinate system of the γ-TuRC. Source data are provided as a Source Data file.

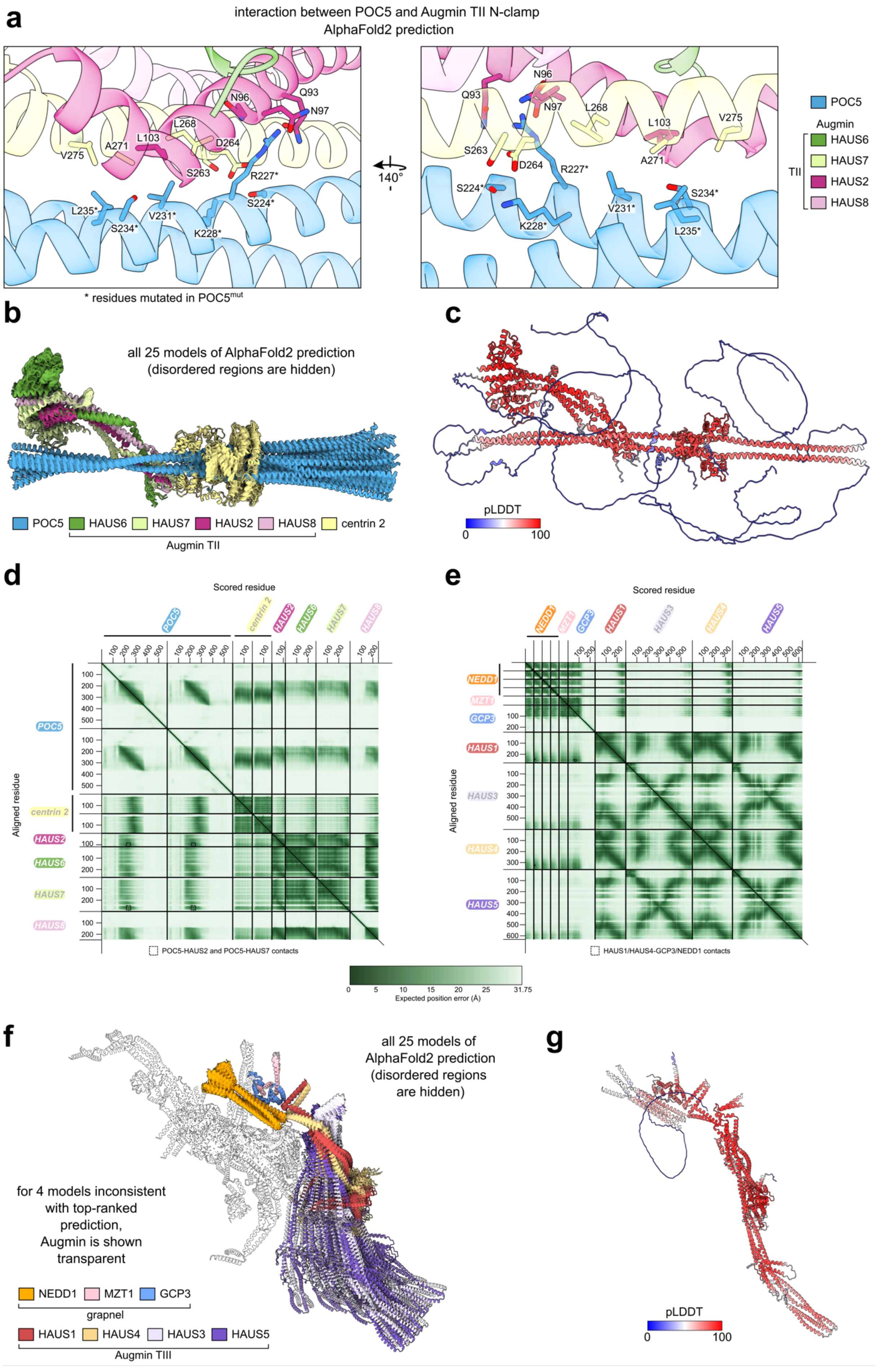

a
interaction between POC5 and Augmin TII N-clamp
AlphaFold2 prediction
N96
Q93
N97
L268
L103
D264
A271
V275
S263
R227*
S224*
L235*
V231*
S234*
K228*
140°
V275
POC5
Augmin
HAUS6
HAUS7
HAUS2
HAUS8
TII
* residues mutated in POC5mut
b
all 25 models of AlphaFold2 prediction
(disordered regions are hidden)
POC5
HAUS6
HAUS7
HAUS2
HAUS8
centrin 2
Augmin TII
c
pLDDT
0
100
d
Scored residue
Aligned residue
POC5
centrin 2
HAUS2
HAUS6
HAUS7
HAUS8
POC5-HAUS2 and POC5-HAUS7 contacts
e
Scored residue
Aligned residue
NEDD1
MZT1
GCP3
HAUS1
HAUS3
HAUS4
HAUS5
HAUS1/HAUS4-GCP3/NEDD1 contacts
Expected position error (Å)
f
all 25 models of
AlphaFold2 prediction
(disordered regions
are hidden)
for 4 models inconsistent
with top-ranked
prediction,
Augmin is shown
transparent
NEDD1
MZT1
GCP3
grapnel
HAUS1
HAUS4
HAUS3
HAUS5
Augmin TIII
g
pLDDT
0
100

**Supplementary Fig. 16. AlphaFold2 predictions of Augmin-POC5 and Augmin-NEDD1 grapnel interactions. a**) Close-up of the interface between POC5 and the Augmin TII N-Clamp predicted by AlphaFold2 (see also panel b-d). Residues mutated in POC5$^{mut}$ are indicated by an asterisk**. b-d**) Full ensemble of 25 models for the AlphaFold2 prediction of the POC5-Augmin TII N-clamp interaction (including POC5-binding protein centrin 2) aligned on the POC5-interacting helix in HAUS7 (b, disordered regions were hidden), top-ranked model including any disordered regions colored by pLDDT (predicted local distance difference test) (c) and the associated PAE (predicted aligned error) matrix (d, dashed boxes indicate interfaces of interest). **e-g**) Full ensemble of 25 models for the AlphaFold2 prediction of the NEDD1 grapnel-Augmin TIII interaction aligned on MZT1 (f, disordered regions were hidden; Augmin is shown transparent for the 4 models inconsistent with the top-ranked model); PAE matrix (e, dashed boxes indicate interfaces of interest), and top-ranked model including any disordered regions colored by pLDDT (g). To reduce prediction complexity, only one N-GCP3/MZT1 module was included in the prediction. Color schemes are indicated. Numbering in PAE matrices starts at the first residue included in the prediction, i.e., not necessarily at the N-terminus of the full-length protein, see also method section. PAE matrices were produced using the PAE Viewer webserver [9]. Source data are provided as a Source Data file.

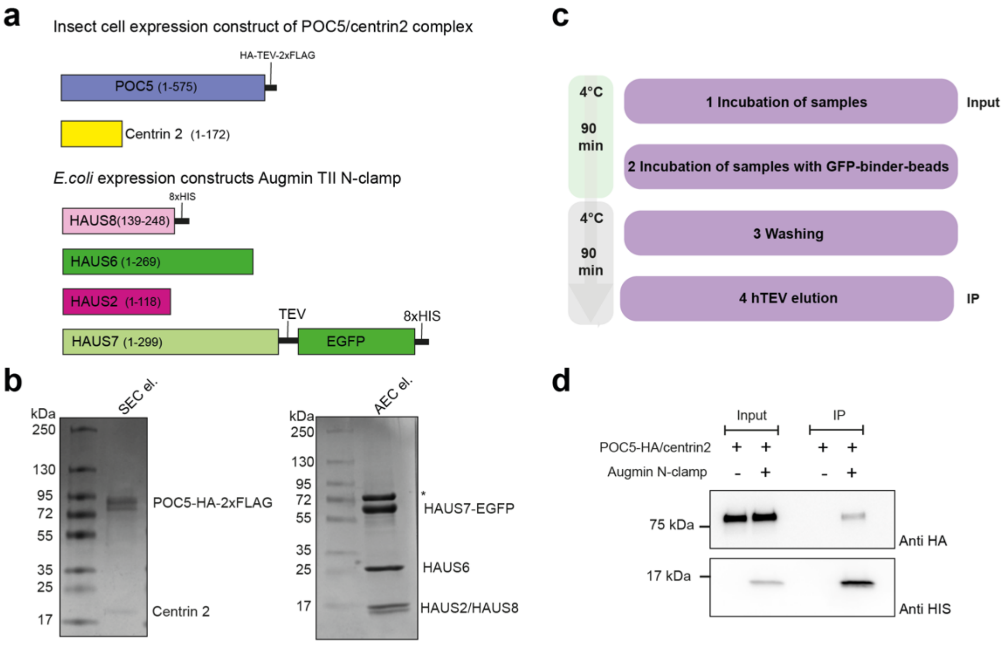


**Supplementary Fig. 17: IP experiments with purified Augmin and POC5 complexes. a)** Expression constructs of the human POC5/centrin 2 complex [18] and the human Augmin TII N-clamp. **b)** Representative section of a Coomassie blue-stained SDS-PAGE for SEC-purified POC5/centrin 2 complex (left) and AEC-purified Augmin TII N-clamp (right). The asterisk indicates an unspecific contaminant. **c)** Schematic workflow of the experiment. Purified Augmin TII N-clamp and POC5/centrin 2 were incubated with GFP-binder beads to capture EGFP-tagged Augmin TII N-clamp (HAUS7). After washing, samples were treated with hTEV protease to cleave Augmin TII N-clamp with bound proteins from the beads. **d)** HAUS7-GFP IP with GFP binder beads. Representative section of immunoblot analysis for the experiment depicted in (c). POC5-HA-2FLAG was detected with anti-HA and Augmin TII with anti-His antibodies (HAUS8$^{139\text{-}248}$-8His). Experiments were repeated with N=3 independent experiments. Source data are provided as a Source Data file.

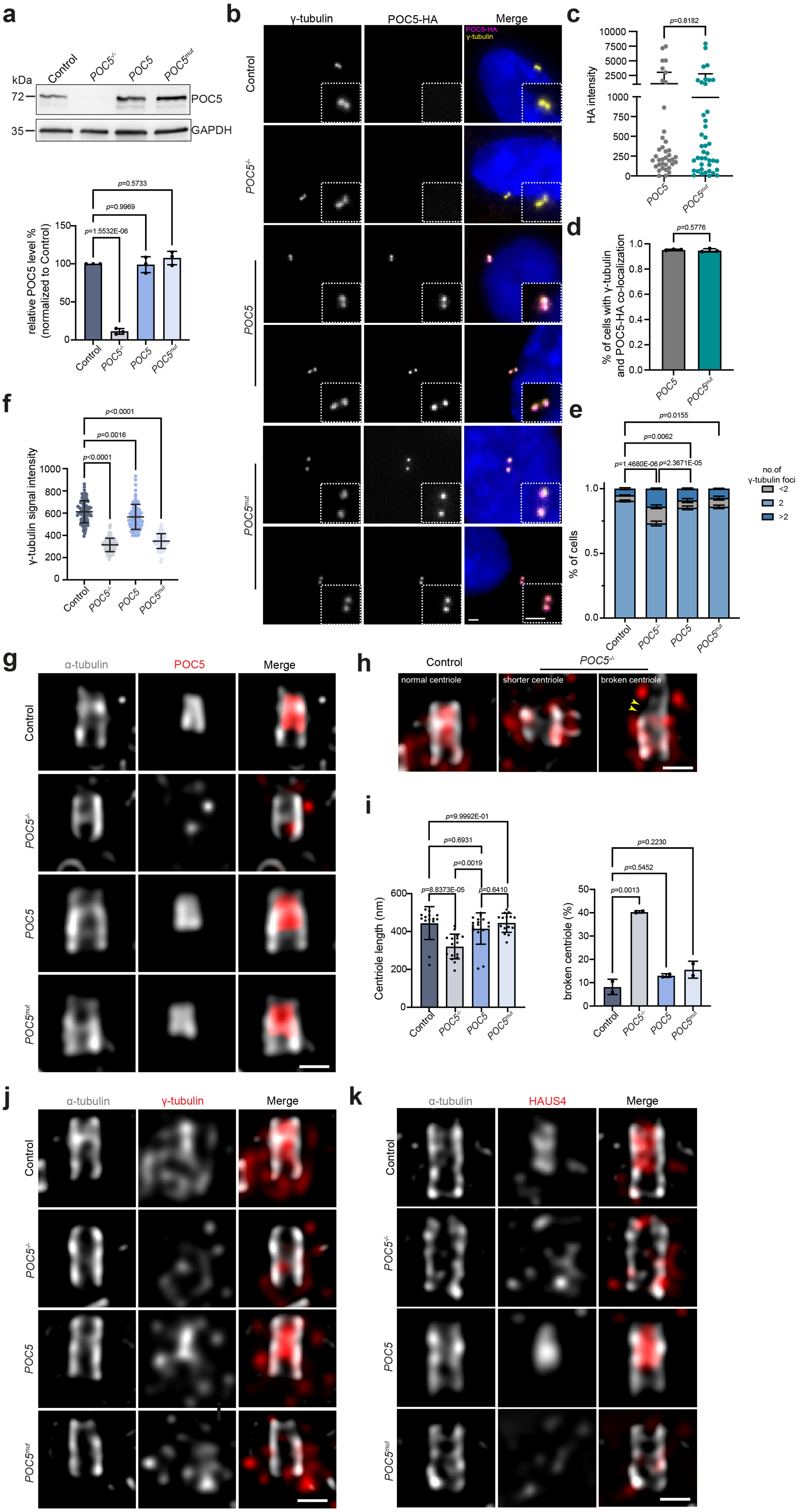

a
Control
POC5-/-
POC5
POC5mut
kDa
72
35
POC5
GAPDH
relative POC5 level % (normalized to Control)
p=0.5733
p=0.9969
p=1.5532E-06
b
γ-tubulin
POC5-HA
Merge
c
HA intensity
p=0.8182
d
% of cells with γ-tubulin and POC5-HA co-localization
p=0.5776
e
% of cells
p=0.0155
p=0.0062
p=1.4680E-06
p=2.3671E-05
no.of γ-tubulin foci
<2
2
>2
f
γ-tubulin signal intensity
p<0.0001
p=0.0016
p<0.0001
g
α-tubulin
Merge
h
normal centriole
shorter centriole
broken centriole
i
Centriole length (nm)
p=9.9992E-01
p=0.6931
p=0.0019
p=8.8373E-05
p=0.6410
broken centriole (%)
p=0.2230
p=0.5452
p=0.0013
j
k
HAUS4

**Supplementary Fig. 18. POC5 is essential for centriole lumenal recruitment of Augmin-γ-TuRC. a)** Upper panel**:** Immunoblot showing protein levels of POC5 in wild-type control, *POC5$^{-/-}$, POC5* and *POC5$^{mut}$* cells. GAPDH was used as loading control. Lower panel**:** Quantification of relative POC5 levels after normalization to GAPDH. **b)** Representative immunofluorescence images of wild type control, *POC5$^{-/-}$*, *POC5-HA* and *POC5$^{mut}$-HA* cells stained against HA (magenta) and the centriolar marker γ-tubulin (yellow). DNA was stained with DAPI (blue). Scale bars: 2 µm. **c)** Quantification (mean ± SD) of HA intensity from *POC5-HA* and *POC5$^{mut}$-HA* cells in panel b. Unpaired two-tailed t-test was applied. N=2 independent experiments. n=38 (*POC5-HA*), 41 (*POC5$^{mut}$-HA*) cells were analyzed. **d)** Quantification (mean ± SD) of panel b, showing the co-localization of γ-tubulin and POC5-HA in most *POC5-HA* and *POC5$^{mut}$-HA* cells (>93%). N=3 independent experiments, n>200 per cell line in total. Unpaired two-tailed t-test was applied. **e)** Quantification of panel b showing percentage of cells with >2, =2 and <2 γ-tubulin foci in *POC5* and *POC5$^{mut}$* cells is comparable. n>80 per cell line were analyzed. **f)** Quantification of γ-tubulin intensity around the centrosome in panel b. n>120 cells were analyzed. **g)** Representative U-ExM images of centrosomes from wild type control, *POC5$^{-/-}$*, *POC5-HA* and *POC5$^{mut}$-HA* cells detected using the indicated antibodies. **h)** Representative U-ExM images of a normal length centriole from a wild type control cell, and a short or broken centriole from *POC5$^{-/-}$* cells. Centrioles were stained against γ-tubulin (red) and $\alpha$-tubulin (grey). Yellow arrows indicate sites of broken centriole MTs. **i)** Left panel: measurement of centriole length from wild type control, *POC5$^{-/-}$*, *POC5-HA* and *POC5$^{mut}$-HA* cells based on U-ExM images. Right panel: ratio of broken centrioles based on U-ExM images. N=2 independent experiments, n>20 centrioles per cell line. Ordinary one-way ANOVA was applied for statistical analysis. **j,k)** Representative U-ExM images of centrosomes from different cells showing impaired centriole localization of HAUS4 and γ-tubulin in *POC5$^{-/-}$* and *POC5$^{mut}$* cells. **g**), **h**), **j**), **k**), scale bars: 200 nm; **a**), **e**), **f**) data are presented as mean ± SD, all statistics were derived from ordinary one-way ANOVA analysis of N=3 biologically independent experiments. Source data are provided as a Source Data file.

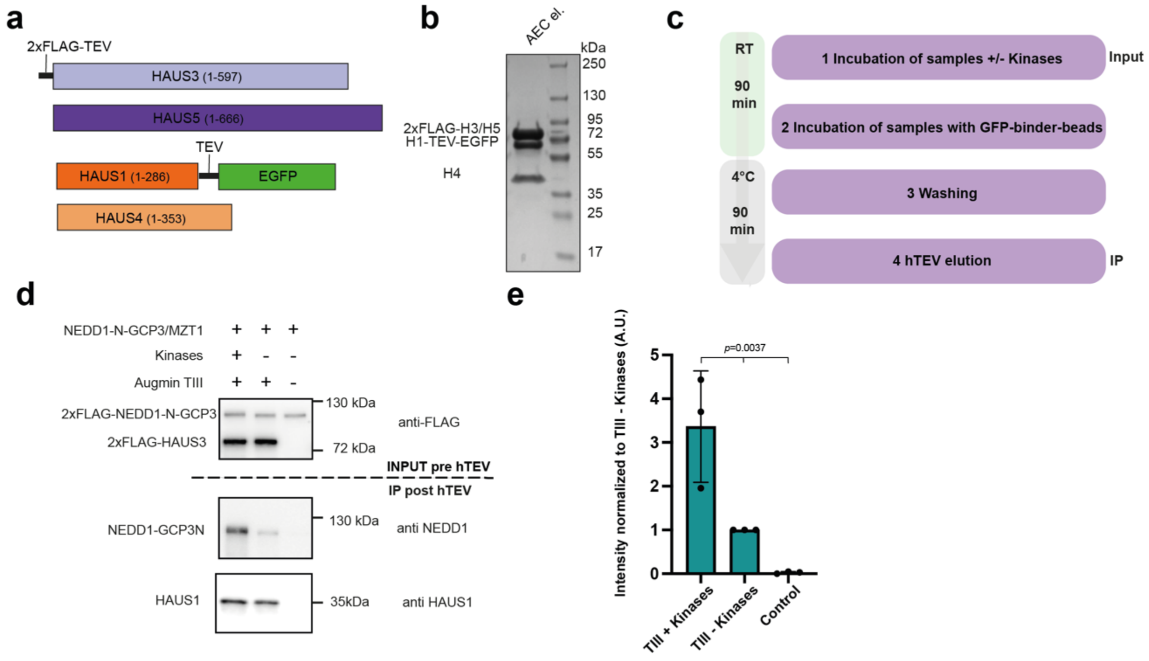


**Supplementary Fig. 19. Augmin TIII directly binds to NEDD1-N-GCP3/MZT1. a)** *X. laevis* Augmin TIII complex insect cell expression construct [19]. **b)** Representative section of an SDS-PAGE gel of AEC-purified Augmin TIII complex. Proteins are labeled as H1 (HAUS1), H3 (HAUS3), H4 (HAUS4), and H5 (HAUS5). **c)** Schematic workflow of the experiment. Purified Augmin TIII and NEDD1-N-GCP3/MZT1 (Supplementary Fig. 4) were incubated either with or without recombinant PLK1 and CDK1/cyclin B1/CKS1 complex. Samples were then incubated with GFP-binder beads to capture EGFP-tagged Augmin TIII (HAUS1). After washing, samples were incubated with hTEV protease to cleave Augmin TIII with bound proteins from the beads. **d)** HAUS1-GFP immunoprecipitation experiment. Representative section of an immunoblot, showing input samples before hTEV incubation using FLAG antibody (top) and eluates after hTEV elution (bottom) using NEDD1 and HAUS1 antibodies. Note that HAUS1 migrates at a lower molecular weight after hTEV digestion compared to panel b due to cleavage from EGFP. Experiments were repeated in N=3 independent replicates. **e)** Quantification (mean ± SD) of band intensity from N=3 independent experiments. Band intensities were normalized to the signal of the condition with Augmin TIII and NEDD1-N-GCP3/MZT1 complex (without kinases). Statistical analysis was done by One-way ANOVA. Source data are provided as a Source Data file.

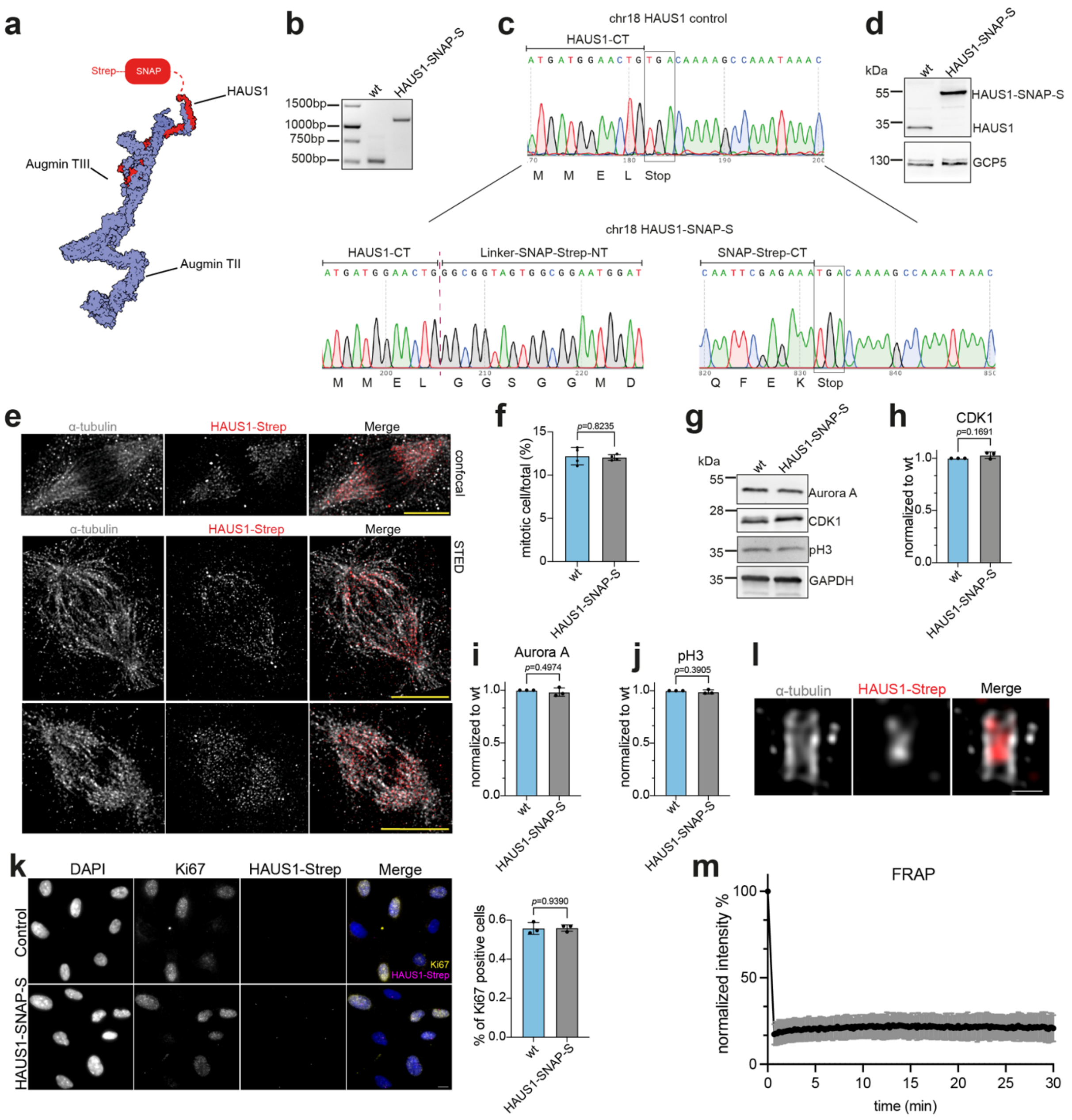


**Supplementary Fig. 20. Verification and characterization of HAUS1-SNAP-S cells. a**) Schematic illustration of Augmin complex and the location of the HAUS1-SNAP-Strep (SNAP-S) tag. **b**) Genotyping PCR of RPE1 wild-type and HAUS1-SNAP-S cells. **c**) Sequencing results of endogenous integration of the SNAP-S tag at the C-terminus of the gene locus of *HAUS1* in RPE1 cells. Top: sequencing result of control cells, stop codon is highlighted. Bottom: sequencing results of *HAUS1-SNAP-S* cells, where on the left the dashed line indicates the former position of *HAUS1* stop codon and on the right the new position on the stop codon following the SNAP-Strep sequence. **d)** Verification of endogenous tagging of HAUS1 with C-terminal SNAP-S tag by immunoblotting with anti-HAUS1 antibody. GCP5 was used as loading control**.** N=2 biologically independent experiments. **e)** Confocal (top) and STED images (two

bottom) of the RPE1 *HAUS1-SNAP-S* cells stained against α-tubulin (gray) and Strep (red). N=2 biologically independent experiments. Scale bars: 5 μm. **f)** Ratio of mitotic cells to the total cells measured by flow cytometry from N=3 independent repeats. **g)** Representative immunoblots showing protein levels of Aurora A, CDK1 and pH3 as mitotic markers, GAPDH was used as the loading control. **h,i,j**) Quantification (mean ± SD) for f) with protein levels of Aurora A, CDK1, and pH3, normalized to GAPDH from wild-type and HAUS1-SNAP-S cells, respectively. N=3 independent experiments. Unpaired two-tailed t-test was applied. **k**) Left**:** representative immunofluorescence images of the control cells and *HAUS1-SNAP-S* cells stained against Ki67 as cell proliferation marker. Scale bars: 5 μm. Right**:** quantification (mean ± SD) of Ki67 signal intensity from control cells and *HAUS1-SNAP* cells from N=3 independent repeats. Unpaired two-tailed t-test was applied. **l)** Confirmation of HAUS1 localization in control cells and *HAUS1-SNAP-S* cells in representative U-ExM images. N=2 biologically independent experiments. Scale bars: 200 nm. **m)** Fluorescence recovery after photobleaching (FRAP) analysis showing no exchange of Augmin in centrioles. SNAP dye (SNAP-Cell TMR-Star dye) labeled HAUS1-SNAP-S was monitored and recorded before and after the photobleaching. The signal was normalized to the level before laser pulse. n>25 samples were analyzed from N=2 independent replicates. Source data are provided as a Source Data file.

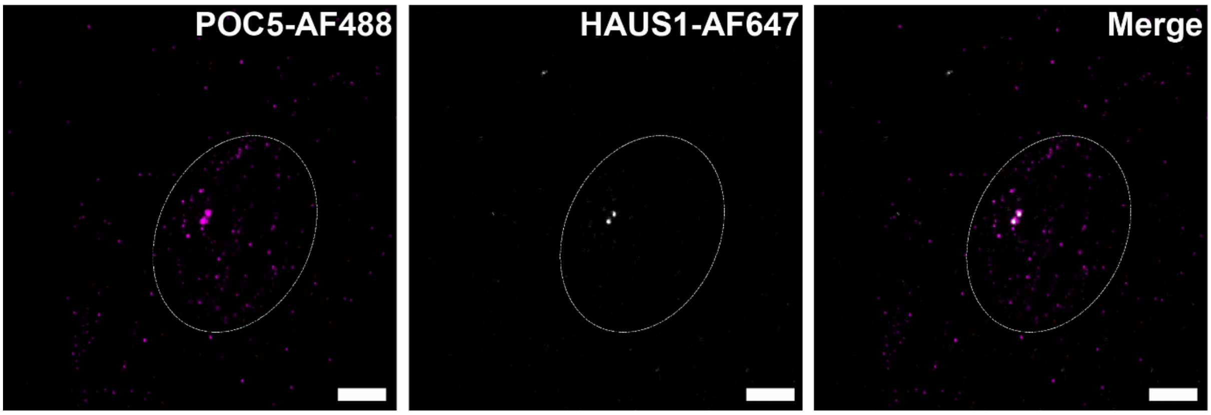


**Supplementary Fig. 21. Confocal overview image of the representative MINFLUX nanoscopy image shown in Figure 5.** Left: POC5 was immunostained by primary and secondary antibody labeling (magenta). The secondary antibody was conjugated to AF488. The white dashed line highlights the area of the cell's nucleus. Middle: HAUS1-SNAP-S-BGAF647 signal (grey). Right: HAUS1-SNAP-S-AF647 and POC5-AF488 signal superposed. N=3 biologically independent experiments. Scale bars: 5 µm.

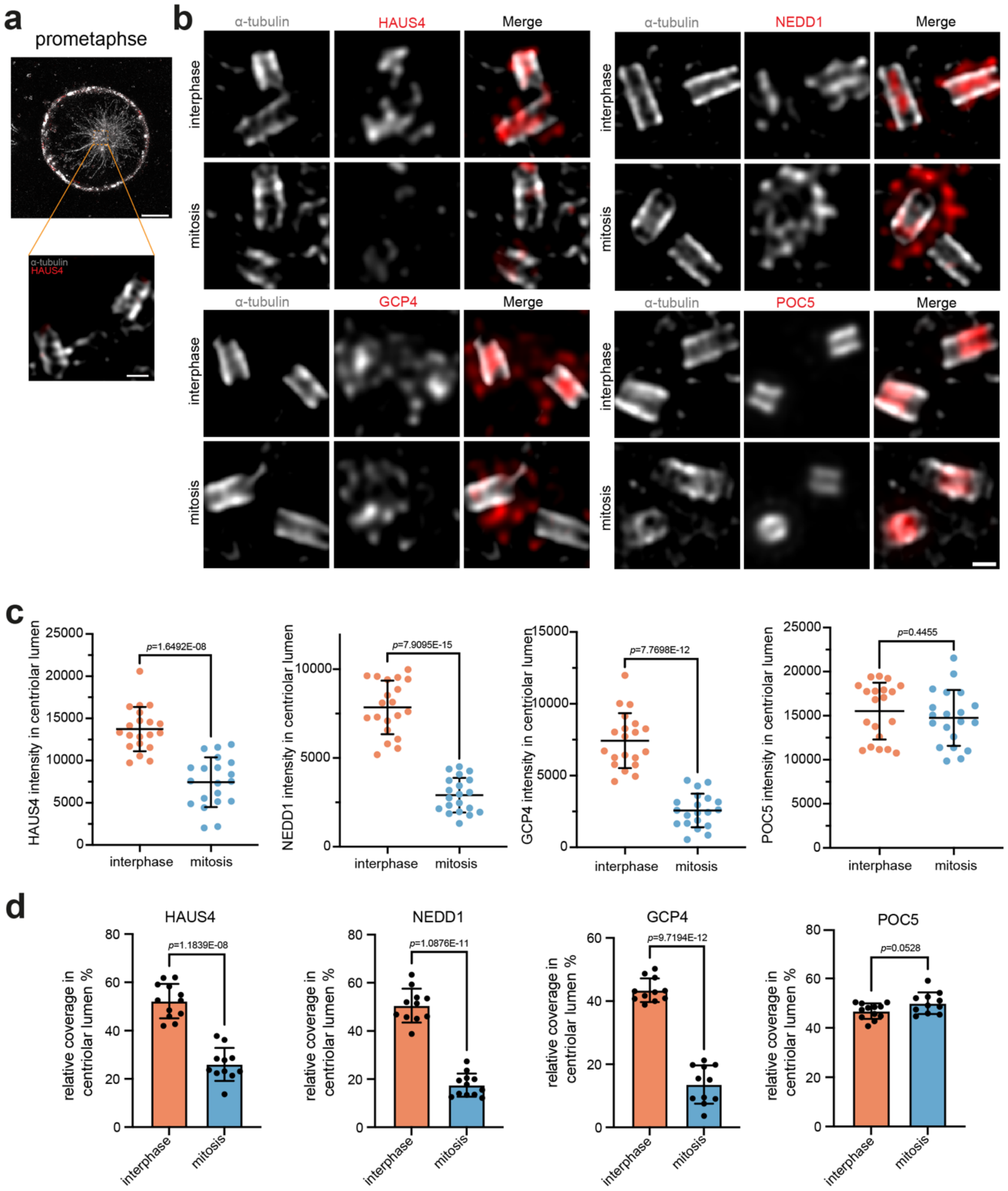


**Supplementary Fig. 22. HAUS4, GCP4 and NEDD1 show reduced centriolar coverage in mitosis. a**) U-ExM image of an RPE1 cell arrested in prometaphase with STLC. MTs (α-tubulin) and Augmin (HAUS4) were stained. The lower panel shows a close-up view of the centrioles. N=3 biologically independent experiments. Scale bar: upper panel 5 µm, lower panel 200 nm. **b)** Representative U-ExM images of RPE1 interphase and mitosis (5 µM STLC treated for 16 hr) cells stained against α-tubulin (grey) and the indicated proteins (red). Scale bar: 200 nm. **c**) Quantification (mean ± SD) of HAUS4, NEDD1, GCP4 and POC5 signal intensity at centrosome during interphase and mitosis from panel b**.** n=20 centrosomes were measured from

N=2 independent experiments per staining condition. Statistical analysis was performed using unpaired two-tailed t-test **d**) Quantification (mean ± SD) of centriolar lumen coverage for indicated proteins in panel b. n>20 samples were included from N=2 independent experiment per staining condition. Statistical analysis was performed using unpaired two-tailed t test. Source data are provided as a Source Data file.

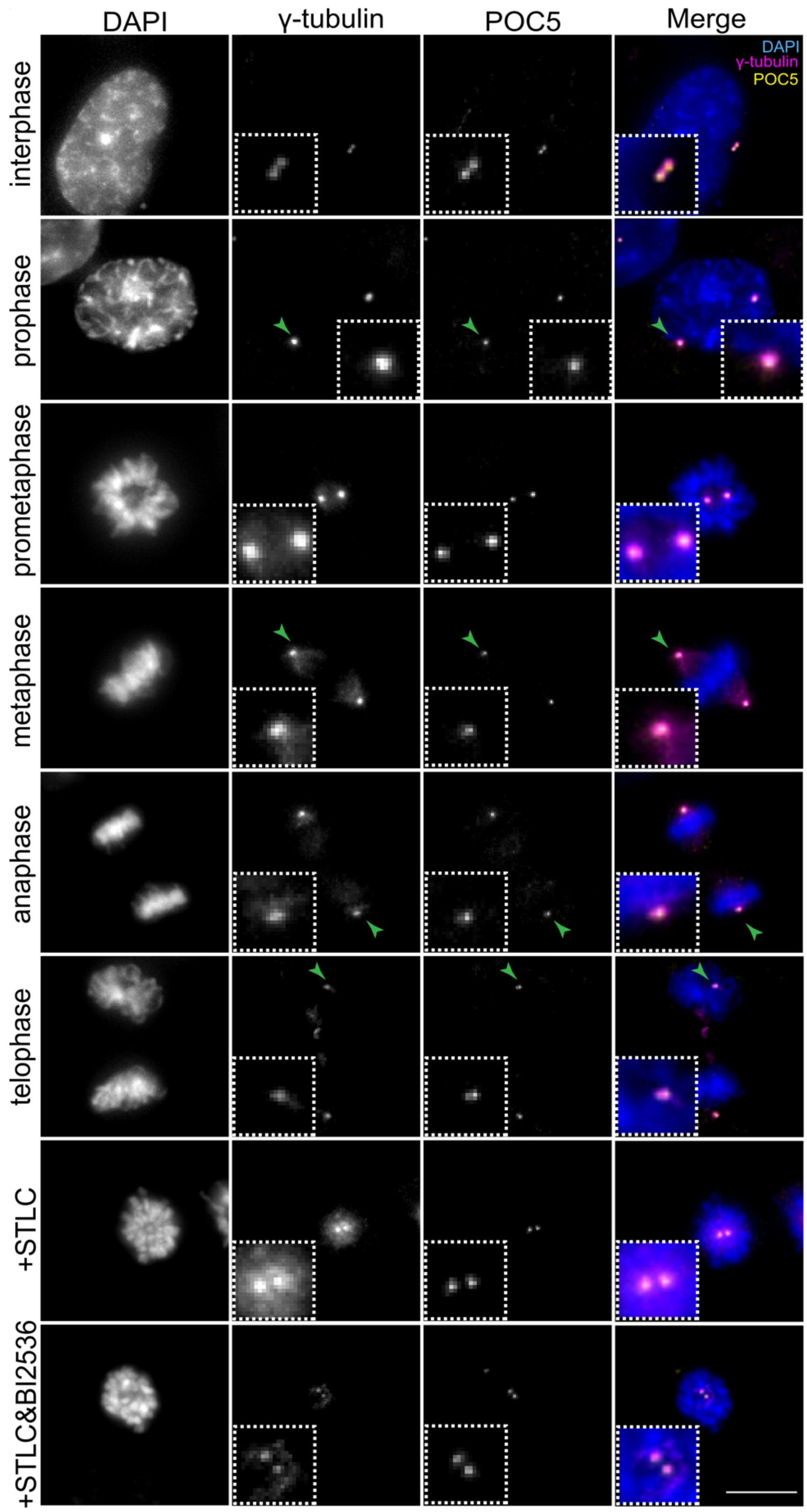


**Supplementary Fig. 23. The localization of the centriole inner scaffold protein POC5 remains constant during the cell cycle.** Representative immunofluorescence images of RPE1 cells in different cell cycle stages stained against the centriolar marker γ-tubulin (magenta) and POC5 (yellow). DNA was visualized with DAPI. Pictures of interphase-telophase cells were taken form an asynchronously growing RPE1 culture.

Cells were treated with STLC for 4 hrs; some other cells were treated with STLC for 4 hrs followed by the PLK1 inhibitor BI2536 for 2 hrs before processing the cells for immunofluorescence. Green arrows indicate centrioles shown in the close-up view. Scale bar: 10 μm.

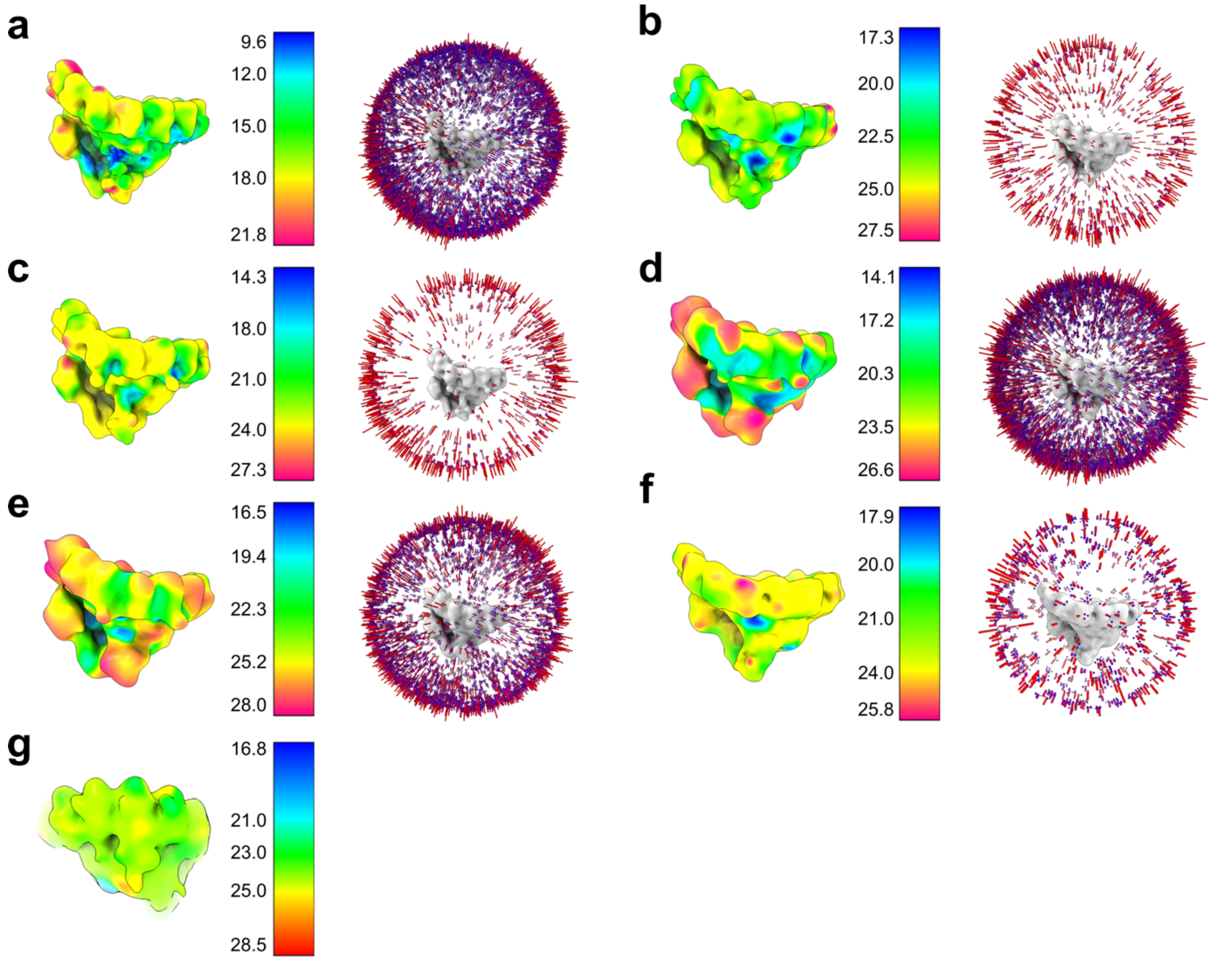


**Supplementary Fig. 24. Local resolution estimation and angular distribution for γ-TuRC reconstructions obtained using cryo-ET. a)** Reconstruction of γ-TuRCs from purified KE37 centrosomes, colored according to local resolution (left) and corresponding angular distribution (right). **b)** Reconstruction of γ-TuRCs in the inwards conformation from purified KE37 centrosomes, colored according to local resolution (left) and corresponding angular distribution (right). **c)** Reconstruction of γ-TuRCs in the outwards conformation from purified KE37 centrosomes, colored according to local resolution (left) and corresponding angular distribution (right). **d)** Reconstruction of γ-TuRCs from purified *wild-type* RPE1 centrosomes, colored according to local resolution (left) and corresponding angular distribution (right). **e)** Reconstruction of γ-TuRCs from purified *CDK5RAP2*$^{-/-}$ RPE1 centrosomes, colored according to local resolution (left) and corresponding angular distribution (right). **f)** Reconstruction of γ-TuRCs in centrosomes of intact human cells, colored according to local resolution (left) and corresponding angular distribution (right). **g)** Reconstruction of γ-TuSC-containing 4-spoked density segment, colored according to local resolution. Angular distribution not available due to extraction of pre-oriented subtomograms from Warp/M.

# Supplementary Tables

**Supplementary Table 1. Mass spectrometry analysis of purified *X. laevis* γ-TuRC used for cryo-EM single particle analysis. The top 40 proteins by total intensity are shown.** γ-TuRC components which were structurally characterized in previous works (blue) and known but yet structurally uncharacterized γ-TuRC components (red) are highlighted.

| Rank | Protein and homeologues |
|---|---|
| 1 | Trypsin |
| 2, 3 | γ-tubulin |
| 4, 5 | GCP3 |
| 6 | GCP2 |
| 7, 8 | TJP3 |
| 9, 27 | MZT2B |
| 10, 11 | ZP3 |
| 12, 13 | NEDD1 |
| 14, 15 | GCP4 |
| 16 | GCP6 |
| 17 | ZP2 |
| 18, 19 | NME7 |
| 20 | GCP5 |
| 21, 22 | ZP4 |
| 23 | HSPA8 |
| 24 | HSPA1 |
| 25, 26 | dynamin GTPase |
| 28,29 | FILIP1 |
| 30 | HSP70 |
| 31, 35 | ZPY1 |
| 32 | Keratin |
| 33 | PPP4R1 |
| 34 | BiP |
| 36, 37, 40 | Actin |
| 38,39 | Neurofibromin |

**Supplementary Table 2. Cryo-EM SPA data collection, refinement and validation statistics.**

| | *X. laevis* γ-TuRC-NEDD1 complex, consensus refinement (PDB: 9I8N / EMD-52730) | *X. laevis* γ-TuRC-NEDD1 complex, focused refinement on grapnel/spoke 9-12 GRIP1 (PDB: 9I8M / EMD-52729) | *X. laevis* γ-TuRC-NEDD1 complex, focused refinement on spokes 12-14 (EMD-52728) |
|---|---|---|---|
| **Data collection and Processing** | | | |
| Microscope | ThermoFisher Titan Krios | ThermoFisher Titan Krios | ThermoFisher Titan Krios |
| Voltage (kV) | 300 | 300 | 300 |
| Camera | Gatan K3 | Gatan K3 | Gatan K3 |
| Magnification | 81,000 | 81,000 | 81,000 |
| Pixel size at detector (Å/pixel) | 1.07 | 1.07 | 1.07 |
| Processing pixel size (Å) | 1.4267 | 1.42945 | 1.4267 |
| Total electron exposure ($e^{-}/Å^2$) | 51 | 51 | 51 |
| Exposure rate ($e^{-}$/pixel/sec) | 15.6 | 15.6 | 15.6 |
| Number of frames collected during exposure | 50 | 50 | 50 |
| Defocus range (μm) | -1 to -3 | -1 to -3 | -1 to -3 |
| Phase plate | - | - | - |
| Automation software | EPU | EPU | EPU |
| Tilt angle | - | - | - |
| Energy filter slid width (eV) | 20 | 20 | 20 |
| Micrographs collected | 29,516 | 29,516 | 29,516 |

| | | | |
|---|---|---|---|
| Micrographs used | 29,516 | 29,516 | 29,516 |
| Total extracted particles | 11,206,035 | 11,206,035 | 11,206,035 |
| **Reconstruction** | | | |
| Final particles | 299,022 | 299,022 | 82,813 |
| Point-group symmetry | C1 | C1 | C1 |
| Resolution (global, Å) | 4.64 | 4.31 | 7.50 |
| FSC threshold | 0.143 | 0.143 | 0.143 |
| Resolution range (Å) | 3.9-23.9 | 3.8-7.1 | 4.7-10.6 |
| Map sharpening B factor (Å$^2$) | -201 | -212 | -285 |
| **Model composition** | | | |
| Protein | 39 | 27 | - |
| Ligands | 0 | 0 | - |
| **Model Refinement** | | | |
| Refinement package | Namdinator | Namdinator | - |
| - Real or reciprocal space | Real space | Real space | - |
| Model composition | | | |
| - Non-hydrogen atoms | 129877 | 37410 | - |
| - Protein residues | 16061 | 4627 | - |
| - Ligands | 0 | 0 | - |
| *B* factors (Å$^2$) | | | |
| - Protein residues (min/max/mean) | 0.10/553.01/166.26 | 15.91/412.88/139.49 | - |
| R.m.s. deviations from ideal values | | | |
| - Bond lengths (Å) | 0.036 | 0.036 | - |
| - Bond angles (°) | 3.43 | 3.48 | - |

| **Model Validation** | | | |
|---|---|---|---|
| MolProbity score | 2.00 | 1.89 | - |
| CaBLAM outliers (%) | 4.85 | 4.84 | - |
| Clashscore | 1.67 | 1.66 | - |
| Poor rotamers (%) | 4.80 | 4.58 | - |
| C-beta deviations (%) | 5.24 | 8.43 | - |
| Ramachandran plot | | | |
| - Favored (%) | 89.51 | 92.31 | - |
| - Outliers (%) | 3.11 | 2.44 | - |

**Supplementary Table 3. Cross correlation analysis indicates that spoke 14 of the native γ-TuRC contains a docked N-GCP5/MZT1 module and NEDD1 interacts with N-GCP3/MZT1 modules.** Crystal structures, cryo-EM- and AlphaFold2 Multimer-derived (Supplementary Fig. 3g-l) atomic models of *Homo sapiens* and *X. laevis* N-GCP3/MZT1 and N-GCP5/MZT1 modules were docked into and cross-correlated against the indicated N-GCP/MZT1 modules as resolved in cryo-EM reconstructions of the *X. laevis* γ-TuRC (EMD 10491 [11]), the consensus reconstruction of the NEDD1-containing *X. laevis* γ-TuRC (this study) and a focused reconstruction of spokes 12-14 of the NEDD1-containing *X. laevis* γ-TuRC with stoichiometric spokes 12-14 (this study, see Supplementary Fig. 5a, Supplementary Fig. 6c-d).

| EM density / Atomic model | | Lumenal bridge | Spoke 14 module | | Grapnel p1 module | Grapnel p2 module | Grapnel d1 module | Grapnel d2 module |
|---|---|---|---|---|---|---|---|---|
| | | EMD 10491 [11] | | This study – consensus refinement | | | | |
| N-GCP3/MZT1 | PDB 6X0U [20] | 0.58 | 0.53 | 0.53 | 0.56 | 0.57 | 0.53 | 0.55 |
| | PDB 7QJD [12] | 0.58 | 0.54 | 0.55 | 0.56 | 0.56 | 0.53 | 0.55 |
| | AF2 | 0.59 | 0.54 | 0.56 | 0.57 | 0.57 | 0.55 | 0.56 |
| N-GCP5/MZT1 | PDB 6L81 [21] | 0.54 | 0.58 | 0.57 | 0.54 | 0.55 | 0.53 | 0.53 |
| | PDB 7QJD [12] | 0.53 | 0.58 | 0.57 | 0.53 | 0.54 | 0.53 | 0.53 |
| Per density color scale: Highest correlation – Lowest correlation | AF2 | 0.54 | 0.58 | 0.58 | 0.54 | 0.53 | 0.53 | 0.53 |

**Supplementary Table 4. Cross correlation analysis indicates N-GCP2/MZT2 modules do not form part of the grapnel or the spoke 14 module.** Crystal structures and AlphaFold2 Multimer-derived (Supplementary Fig. 3g-l, Supplementary Fig. 7a-c) atomic models of *H. sapiens* and *X. laevis* N-GCP2/MZT2 modules were docked into and cross-correlated against the indicated N-GCP/MZT modules as resolved in cryo-EM reconstructions of the *X. laevis* γ-TuRC (EMD 10491 [11]), the human CM1 motif-containing γ-TuRC (EMD 21985 [20]), the consensus reconstruction of the NEDD1-containing *X. laevis* γ-TuRC (this study) and a focused reconstruction of spokes 12-14 of the NEDD1-containing *X. laevis* γ-TuRC (this study, see Supplementary Fig. 5a, Supplementary Fig. 6c-d). The cross-correlation values for N-GCP2/MZT2 modules were compared to the best scoring N-GCP/MZT1 modules from Supplementary Table 3.

| Atomic model \ EM density | | Lumenal bridge | Spoke 14 module | | Grapnel p1 module | Grapnel p2 module | Grapnel d1 module | Grapnel d2 module | N-GCP2/MZT2 module |
|---|---|---|---|---|---|---|---|---|---|
| | | EMD: 10491 [11] | | This study – consensus refinement | | | | | EMD: 21985 |
| N-GCP3/MZT1 | PDB: 6X0U [20] | 0.58 | | | 0.56 | 0.57 | 0.53 | 0.55 | 0.37 |
| | PDB: 7QJD [12] | 0.58 | | | 0.56 | 0.56 | 0.53 | 0.55 | 0.39 |
| | AF2 | 0.59 | | | 0.57 | 0.57 | 0.55 | 0.56 | 0.38 |
| N-GCP5/MZT1 | PDB: 6L81 [21] | | 0.58 | 0.57 | | | | | 0.38 |
| | PDB: 7QJD [12] | | 0.58 | 0.57 | | | | | 0.37 |
| | AF2 | | 0.58 | 0.58 | | | | | 0.34 |
| N-GCP2/MZT2 | PDB: 7FAD | 0.50 | 0.52 | 0.51 | 0.50 | 0.52 | 0.51 | 0.52 | 0.55 |
| | AF2 | 0.51 | 0.51 | 0.52 | 0.51 | 0.51 | 0.51 | 0.52 | 0.54 |

Per density color scale: Highest correlation – Lowest correlation

**Supplementary Table 5. MINFLUX parameters used in the analysis.** The table provides quality parameters, the localization precision (σ), the center frequency ratio (cfr) and the effective frequency at offset (efo) of each MINFLUX data set used for further data analysis. The localization precision is the standard deviation calculated from traces with at least four valid localizations. The cfr values are obtained from the sixth iteration of the MINFLUX sequence. The efo values are obtained from the ninth iteration of the valid localizations. Source data are provided as a Source Data file.

| Data set [date-time] | Median σ_x [nm] | median σ_y [nm] | median σ_z [nm] | median cfr | median efo [kHz] |
|---|---|---|---|---|---|
| 231030-165937_minflux | 6 | 6.3 | 6.4 | 0.34 | 21.5 |
| 231031-105705_minflux | 5.4 | 6 | 4.2 | 0.25 | 22.33 |
| 231031-153606_minflux | 6 | 7.1 | 4.6 | 0.35 | 22.33 |
| 231116-143030_minflux | 6 | 6.1 | 4.9 | 0.26 | 22.33 |
| 231116-153654_minflux | 5.8 | 5.6 | 4.6 | 0.37 | 24.67 |
| 231116-161243_minflux | 6 | 5.6 | 5.2 | 0.29 | 24.33 |
| 231117-092006_minflux | 6.8 | 6.6 | 5.4 | 0.42 | 30.5 |
| 231117-095700_minflux | 6.7 | 6.7 | 4.3 | 0.52 | 31.5 |
| 231117-115308_minflux | 6.5 | 6.5 | 5.3 | 0.42 | 28.5 |
| 231117-144400_minflux | 6.7 | 6.1 | 5.1 | 0.45 | 27.5 |
| 231117-152826_minflux | 6 | 5.6 | 4.5 | 0.4 | 25 |
| 231117-160557_minflux | 5.9 | 6.1 | 5.4 | 0.29 | 25 |

| | | | | | |
|---|---|---|---|---|---|
| 231123-113138_minflux | 7.4 | 6.6 | 4.7 | 0.46 | 27.5 |
| 231123-123107_minflux | 6.6 | 6.6 | 5.5 | 0.4 | 27 |
| 231123-132324_minflux | 5.9 | 5.7 | 4.8 | 0.35 | 25.33 |
| 231123-135456_minflux | 6.2 | 6 | 3.8 | 0.51 | 29.5 |
| 231215-132439_minflux | 5.9 | 5.9 | 3.8 | 0.48 | 29.5 |

**Supplementary Table 6. AlphaFold prediction details.** Sequence fragments, corresponding UniProt IDs, AlphaFold version and relaxation status used for different predictions presented in this manuscript. AlphaFold predictions used as initial models for model building are marked accordingly. * Model was trimmed to maintain only the interface of interest due to memory constraints. ** Residues 238-249 and 250-262 were mutated to poly-A and poly-GS, respectively, to avoid prediction of the N-GCP3/MZT1-GCP5 interface at spoke 10. *** Prediction was retrieved from https://alphafold.ebi.ac.uk/entry/A0A1L8HGZ5. FL = full length.

| Predicted proteins (residue range; number of copies) | Organism | UniProt IDs | AlphaFold version | Relaxation | Used for model building | ModelArchive ID |
|---|---|---|---|---|---|---|
| NEDD1 (585-660; 4), GCP3 (1-245; 4), MZT1 (FL; 4) | *H. sapiens* | Q8NHV4, Q96CW5, Q08AG7 | 2.3.2 | yes | no | ma-odixd |
| NEDD1 (566-671; 4), GCP3 (1-126; 4), MZT1 (FL; 4) | *X. laevis* | A0A8J0Q0Y8, O73787, Q5U4M5 | 2.3.2 | yes | yes | ma-imf31 |
| GCP3 (1-101; 1), MZT1 (FL; 1) | *X. laevis* | O73787, Q5U4M5 | 2.3.2 | yes | no | ma-14965 |
| GCP5 (1-114; 1), MZT1 (FL; 1) | *X. laevis* | A0A1L8HGZ5, Q5U4M5 | 2.3.2 | yes | no | ma-yzz9w |

| | | | | | | |
|---|---|---|---|---|---|---|
| GCP2 (1-107; 1), MZT2B (44-93; 1) | *X. laevis* | A0A8J0T6B8, A0A974DV87 | 2.3.2 | yes | no | ma-8bwwq |
| GCP2 (129-508; 1), GCP3 (245-544; 1), GCP6 (184-277; 1) | *X. laevis* | A0A8J0T6B8, O73787, A0A974HT83 | 2.3.2 | yes | yes | ma-1vc4y |
| POC5 (FL; 2), centrin 2 (FL; 2), HAUS2 (1-118; 1), HAUS6 (1-268; 1), HAUS7 (1-299; 1), HAUS8 (1-249; 1) | *H. sapiens* | Q8NA72, P41208, Q9NVX0, Q7Z4H7, Q99871, Q9BT25 | 2.3.1 | top-ranked model* | no | ma-rwmkh |
| NEDD1 (585-660; 4), GCP3 (1-245; 1), MZT1 (FL; 1), HAUS1 (FL; 1), HAUS3 (FL; 1), HAUS4 (FL; 1), HAUS5 (FL; 1) | *H. sapiens* | Q8NHV4, Q96CW5, Q08AG7, Q96CS2, Q68CZ6, Q9H6D7, O94927 | 2.3.1 | no | no | ma-89x6h |
| GCP3 (1-126; 1), MZT1 (FL; 1), GCP4 | *X. laevis* | O73787, Q5U4M5, Q642S3, A0A1L8HGZ5 | 2.3.2 | yes | yes | ma-rmctb |

| | | | | | | |
|---|---|---|---|---|---|---|
| (1-340; 1), GCP5 (164-699; 1) | | | | | | |
| GCP3 (1-126; 1), MZT1 (FL; 1), GCP4 (1-340; 1), GCP5 (164-699**; 1), GCP6 (303-658 fused to 1324-1387; 1) | *X. laevis* | O73787, Q5U4M5, Q642S3, A0A1L8HGZ5, A0A974HT83 | 2.3.2 | yes | yes | ma-phn3m |
| GCP6 (FL; 1) | *X. laevis* | A0A974HT83 | 2.3.1 | no | yes | ma-z68wf |
| GCP5 (FL; 1) | *X. laevis* | A0A1L8HGZ5 | 2.0 *** | yes | yes | - |
| CDK5RAP2 (60-200; 2), GCP2 (FL; 1), GCP3 (FL; 1), γ-tubulin (FL; 2), MZT2A (FL; 1) | *H. sapiens* | Q96SN8, Q9BSJ2, Q96CW5, P23258, Q6P582 | 3 | yes | no | ma-2zl3w |

## Supplementary Table 7. Primer List

| Name | Sequence (5’ → 3’) | Source | Identifier |
|---|---|---|---|
| PL_1stside_f | TGAGAATTCGAGCTCGGC | This study | N/A |
| PL_1stside_r | CATGGTATATCTCCTTCTTAAAGTTAAAC | This study | N/A |
| PL_2ndside_f | CTGTCCGGCCATCATCAC | This study | N/A |
| PL_2ndside_r | CATATGTATATCTCCTTCTTATACTTAACTAATATACTAAG | This study | N/A |
| Haus2_f | taagaaggagatataccatgGCCGCTGCCAACCCGTGG | This study | N/A |
| Haus2_r | gcgccgagctcgaattctcaGCACATGGGTTTCAACAGTCTTTGCCTAAG | This study | N/A |
| Haus7_f | aagaaggagatatacatatgGCGGGGCAGGACGCTGGC | This study | N/A |
| Haus7_r | tggtgatgatggccggacagCTGGATGATGGGGCCGCACG | This study | N/A |
| Haus6_f | taagaaggagatataccatgAGCTCGGCCTCGGTCACC | This study | N/A |
| Haus6_r | gcgccgagctcgaattctcaTCCATCTAAAGCATATTGGTTAACAAGACTAAGGACC | This study | N/A |
| Haus8_f | aagaaggagatatacatatgGCGGATTCCTCGGGGCGAG | This study | N/A |
| Haus8_r | tggtgatgatggccggacagGGGCAGCTCGTGCCTGGT | This study | N/A |
| Pet26-EGFP_conversion_PL _f | ccatcatccagctgtccggcGAAAACCTGTATTTTCAGGGC | This study | N/A |
| Pet26-EGFP_conversion_PL _r | TCACCGATGGGGAAGATC | This study | N/A |
| Pet26-EGFP_conversion_insert_f | ccgatcttccccatcggtgaCCGGCGTAGAGGATCGAG | This study | N/A |
| Pet26-EGFP_conversion_insert_r | GCCGGACAGCTGGATGATG | This study | N/A |
| HAUS8_deltN_f | aagaaggagatatacatatgAGCCCGGATTTATCTGAAG | This study | N/A |
| HAUS8_deltN_r | CATATGTATATCTCCTTCTTATACTTAAC | This study | N/A |
| Pet26PL_f | ccatcatccagctgtccggcGAAAACCTGTATTTTCAGGGC | This study | N/A |
| Pet26PL_r | TCACCGATGGGGAAGATC | This study | N/A |
| Duet_inser_f | CCGATCTTCCCCATCGGTG | This study | N/A |
| Duet_inser_r | GCCGGACAGCTGGATGATG | This study | N/A |
| pRetroX-Tet3G_f | GGTGGCGCACGCGTATCG | This study | N/A |
| pRetroX-Tet3G_r | GGAGCTGGTGCAGGTGCAG | This study | N/A |
| NEDD1$^{VVI}$ f | GAAGCATGCCATAGGGACATTGCGAATTTGCAAGCGGAGATGGCTAAACAGTTTCATATGCAACTGAATGA | This study | N/A |
| NEDD1$^{VVI}$ r | GTCCCTATGGCATGCTTC | This study | N/A |
| NEDD1$^{LLM}$ f | TGATACAGGAAACGGACGATGACTTTAGAGAAGCATGCCATAGGGACATTGTGAATGACCAAGTGGAGATGATTAAACAGTTTCATATGCAACTGAATGAAGACCATTCTTTGCTGGAA | This study | N/A |
| NEDD1$^{LLM}$ r | CGTTTCCTGTATCATGTTCTG | This study | N/A |
| pRetroX-Tet3G-NEDD1PL-f | CTACCGGGTAGGGGAGGCGC | This study | N/A |
| pRetroX-Tet3G-NEDD1PL-r | GGTGGCGCACGCGTATCG | This study | N/A |

| | | | |
|---|---|---|---|
| NEDD1- pRetroX_in_f | ATCGATACGCGTGCGCCACCATGTACCCATACGATGTTCCAGATTACGCTGGAGCTGGTGCAGGTGCAGGAGCTGGTGCAATGCAGGAAAACCTCAGATTTGCTTC | This study | N/A |
| NEDD1- pRetroX_in_r | GCGCCTCCCCTACCCGGTAGTCAAAAGTGGGCCCGTAATC | This study | N/A |
| POC5-PL_f | GGAGCTGGTGCAGGTGCAG | This study | N/A |
| POC5-PL_r | GGTGGCGCACGCGTATCG | This study | N/A |
| Multibac_f | TCTAGAGCCTGCAGTCTCG | (Würtz et al) | N/A |
| Multibac_r | ATATTTATAGGTTTTTTTATTACAAAACTG | (Würtz et al) | N/A |
| 2xFlag-pacebac1 _f | TCTAGAGCCTGCAGTCTC | This study | N/A |
| 2xFlag-pacebac1 _r | GGCGCCCTGAAAATACAG | This study | N/A |
| XL_Nedd1_f | acctgtattttcagggcgccGGCGGCATGCAGGATAAC | This study | N/A |
| XL_Nedd1_r | tcgagactgcaggctctagaCTAAAAATTGGCCCGCAATC | This study | N/A |
| NEDD1_fusion_PL_f | TCGACAAGCTTGTCGAGAAG | This study | N/A |
| NEDD1_fusion_PL_r | AAAATTGGCCCGCAATCTTTTATTC | This study | N/A |
| POC5-insert_f | ATCGATACGCGTGCGCCACCATGTCATCAGATGAGGAG | This study | N/A |
| POC5-insert_r | CCTGCACCTGCACCAGCTCCGTCAACCACTTTTATGGAATG | This study | N/A |
| POC5-mutPL_f | ATAGGCAAGCAAAAGGAAAAG | This study | N/A |
| POC5-mutPL_r | TTCAAAGGTTTTTTGCAGC | This study | N/A |
| HAUS1SNAP_genotyping_f | cagagattgcagagtgag | This study | N/A |
| HAUS1SNAP_genotyping_r | ggtgtgaagtatggacct | This study | N/A |

**Supplementary Table 8. Cryo-ET data collection and refinement statistics.** Wherever indicated, data acquisition parameters for cellular tomograms from HeLa cells are as in Li et al. [17].

| **Data collection and Processing** | KE37 γ-TuRC - consensus refinement (EMD-52717) | KE37 γ-TuRC - inwards conformation (PDB-9I8G/ EMD-52718) | KE37 γ-TuRC - outwards conformation (PDB- 9I8H/ EMD-52719) | HCT116/Hela γ-TuRC - cellular tomography (EMD-52722) | HCT116/Hela MTT - cellular tomography (EMD-52723) | RPE1 wt γ-TuRC (EMD-52721) | RPE1 CDK5RAP2$^{-/-}$ γ-TuRC (EMD-52720) |
|---|---|---|---|---|---|---|---|
| Microscope | ThermoFisher Titan Krios | ThermoFisher Titan Krios | ThermoFisher Titan Krios | ThermoFisher Titan Krios / *Li et al.* | *Li et al.* | ThermoFisher Titan Krios | ThermoFisher Titan Krios |
| Voltage (kV) | 300 | 300 | 300 | 300 / *Li et al.* | *Li et al.* | 300 | 300 |
| Camera | Gatan K3 | Gatan K3 | Gatan K3 | Gatan K3 / *Li et al.* | *Li et al.* | Gatan K3 | Gatan K3 |
| Magnification | 33 000 | 33 000 | 33 000 | 33 000 / *Li et al.* | *Li et al.* | 33 000 | 33 000 |
| Pixel size at detector (Å/pixel) | 2.54 | 2.54 | 2.54 | 2.54 / Li et al. | Li et al. | 2.54 | 2.54 |
| Processing pixel size (Å) | 2.54 | 5.08 | 5.08 | 4.3 / Li et al. | 8.6 | 5.08 | 5.08 |
| Total electron exposure (e$^-$/Å$^2$) | 217.3 | 217.3 | 217.3 | 135.3 / Li et al. | *Li et al.* | 225.5 | 225.5 |
| Exposure rate (e$^-$/pixel/sec) | 27.333 | 27.333 | 27.333 | 31.37 / *Li et al.* | *Li et al.* | 26.358 | 26.358 |
| Number of frames collected during exposure | 5 | 5 | 5 | 5 / *Li et al.* | *Li et al.* | 5 | 5 |
| Defocus range (μm) | -3 to -6 | -3 to -6 | -3 to -6 | *-6 / Li et al* | *Li et al.* | -4 to -5 | -4 to -5 |
| Automation software | EPU Tomography | EPU Tomography | EPU Tomography | SerialEM / *Li et al.* | *Li et al.* | EPU Tomography | EPU Tomography |
| Energy filter slid width (eV) | 20 | 20 | 20 | 20 / *Li et al.* | *Li et al.* | 20 | 20 |
| Tilt-series used | 50 | 50 | 50 | 2 / 12 | 12 | 19 | 21 |
| Total extracted particles | 65786 | 65786 | 65786 | 8260 | 2040 | 21361 | 25014 |
| **Reconstruction** | | | | | | | |
| Final particles | 4402 | 780 | 739 | 57 | 2040 | 3931 | 2848 |
| Point-group symmetry | C1 | C1 | C1 | C1 | C1 | C1 | C1 |
| Resolution (global. Å) | 16.4 | 22.4 | 23.2 | 30.6 | 30.5 | 29.6 | 30.4 |
| FSC threshold | 0.143 | 0.143 | 0.143 | 0.143 | 0.143 | 0.143 | 0.143 |

## Supplementary References


1 Zheng, S. Q. *et al.* MotionCor2: anisotropic correction of beam-induced motion for improved cryo-electron microscopy. *Nature Methods* **14**, 331-332 (2017). https://doi.org/10.1038/nmeth.4193
2 Tegunov, D. & Cramer, P. Real-time cryo-electron microscopy data preprocessing with Warp. *Nature Methods* **16**, 1146-1152 (2019). https://doi.org/10.1038/s41592-019-0580-y
3 Kremer, J. R., Mastronarde, D. N. & McIntosh, J. R. Computer visualization of three-dimensional image data using IMOD. *J Struct Biol* **116**, 71-76 (1996). https://doi.org/10.1006/jsbi.1996.0013
4 Mastronarde, D. N. Dual-Axis Tomography: An Approach with Alignment Methods That Preserve Resolution. *Journal of Structural Biology* **120**, 343-352 (1997). https://doi.org/https://doi.org/10.1006/jsbi.1997.3919
5 Hrabe, T. *et al.* PyTom: A python-based toolbox for localization of macromolecules in cryo-electron tomograms and subtomogram analysis. *Journal of Structural Biology* **178**, 177-188 (2012). https://doi.org/https://doi.org/10.1016/j.jsb.2011.12.003
6 Zivanov, J., Nakane, T. & Scheres, S. H. W. Estimation of high-order aberrations and anisotropic magnification from cryo-EM data sets in RELION-3.1. *IUCrJ* **7**, 253-267 (2020). https://doi.org/10.1107/s2052252520000081
7 Bharat, T. A. M. & Scheres, S. H. W. Resolving macromolecular structures from electron cryo-tomography data using subtomogram averaging in RELION. *Nature Protocols* **11**, 2054-2065 (2016). https://doi.org/10.1038/nprot.2016.124
8 Tegunov, D., Xue, L., Dienemann, C., Cramer, P. & Mahamid, J. Multi-particle cryo-EM refinement with M visualizes ribosome-antibiotic complex at 3.5 Å in cells. *Nature Methods* **18**, 186-193 (2021). https://doi.org/10.1038/s41592-020-01054-7
9 Elfmann, C. & Stülke, J. PAE viewer: a webserver for the interactive visualization of the predicted aligned error for multimer structure predictions and crosslinks. *Nucleic Acids Research* **51**, W404-W410 (2023). https://doi.org/10.1093/nar/gkad350
10 Kidmose, R. T. *et al.* Namdinator - automatic molecular dynamics flexible fitting of structural models into cryo-EM and crystallography experimental maps. *IUCrJ* **6**, 526-531 (2019). https://doi.org/10.1107/s2052252519007619
11 Liu, P. *et al.* Insights into the assembly and activation of the microtubule nucleator γ-TuRC. *Nature* **578**, 467-471 (2020). https://doi.org/10.1038/s41586-019-1896-6
12 Würtz, M. *et al.* Modular assembly of the principal microtubule nucleator γ-TuRC. *Nat Commun* **13**, 473 (2022). https://doi.org/10.1038/s41467-022-28079-0
13 Wieczorek, M. *et al.* Asymmetric Molecular Architecture of the Human γ-Tubulin Ring Complex. *Cell* **180**, 165-175.e116 (2020). https://doi.org/10.1016/j.cell.2019.12.007
14 Goddard, T. D. *et al.* UCSF ChimeraX: Meeting modern challenges in visualization and analysis. *Protein Sci* **27**, 14-25 (2018). https://doi.org/10.1002/pro.3235
15 Meng, E. C. *et al.* UCSF ChimeraX: Tools for structure building and analysis. *Protein Science* **32**, e4792 (2023). https://doi.org/https://doi.org/10.1002/pro.4792
16 Pettersen, E. F. *et al.* UCSF ChimeraX: Structure visualization for researchers, educators, and developers. *Protein Sci* **30**, 70-82 (2021). https://doi.org/10.1002/pro.3943
17 Li, S. *et al.* ELI trifocal microscope: a precise system to prepare target cryo-lamellae for in situ cryo-ET study. *Nature Methods* **20**, 276-283 (2023). https://doi.org/10.1038/s41592-022-01748-0

18 Sala, C. *et al.* An interaction network of inner centriole proteins organised by POC1A-POC1B heterodimer crosslinks ensures centriolar integrity. *Nature Communications* **15**, 9857 (2024). https://doi.org/10.1038/s41467-024-54247-5

19 Zupa, E. *et al.* The augmin complex architecture reveals structural insights into microtubule branching. *Nature Communications* **13**, 5635 (2022). https://doi.org/10.1038/s41467-022-33228-6

20 Wieczorek, M., Huang, T.-L., Urnavicius, L., Hsia, K.-C. & Kapoor, T. M. MZT Proteins Form Multi-Faceted Structural Modules in the γ-Tubulin Ring Complex. *Cell Reports* **31**, 107791 (2020). https://doi.org/https://doi.org/10.1016/j.celrep.2020.107791

21 Huang, T. L., Wang, H. J., Chang, Y. C., Wang, S. W. & Hsia, K. C. Promiscuous Binding of Microprotein Mozart1 to γ-Tubulin Complex Mediates Specific Subcellular Targeting to Control Microtubule Array Formation. *Cell Rep* **31**, 107836 (2020). https://doi.org/10.1016/j.celrep.2020.107836
