# Supplementary material for: Structural mechanisms for centrosomal recruitment and organization of the microtubule nucleator γ-TuRC
Source: Nat Commun. 2025 Mar 12;16:2453. doi: 10.1038/s41467-025-57729-2 (PMC11903878; doi:10.1038/s41467-025-57729-2)
Supplement: Supplementary file 2 — Description of Additional Supplementary Files [file 41467_2025_57729_MOESM2_ESM.pdf]

Description of Additional Supplementary Files

File name: Supplementary Data 1
Description: Mass Spectrometry Data for γ-TuRC purified from *Xenopus laevis* egg extract.

File name: Supplementary Software 1
Description: Custom scripts used in this study.
