# Supplementary material for: Structural mechanisms for centrosomal recruitment and organization of the microtubule nucleator γ-TuRC
Source: Nat Commun. 2025 Mar 12;16:2453. doi: 10.1038/s41467-025-57729-2 (PMC11903878; doi:10.1038/s41467-025-57729-2)
Supplement: Supplementary file 4 — Supplementary Software 1 [file 41467_2025_57729_MOESM4_ESM.zip › Scripts/TableS2_MINFLUX/231123_example_processed/231123_acquisition_info.pdf]

231123-123107_minflux.csv:

Number of localizations: 1476

Number of traces: 82

Locs to trace ratio: 18.0 : 1

Time of acquisition: 0.49 h

Mean drift_x: -2.3 nm

Median drift_x: -1.9 nm

Mean drift_y: 0.8 nm

Median drift_y: 0.4 nm

Mean drift_z: -3.8 nm

Median drift_z: -4.1 nm

mock
