# Supplementary figures and images for: Structural mechanisms for centrosomal recruitment and organization of the microtubule nucleator γ-TuRC
Source: Nat Commun. 2025 Mar 12;16:2453. doi: 10.1038/s41467-025-57729-2 (PMC11903878; doi:10.1038/s41467-025-57729-2)

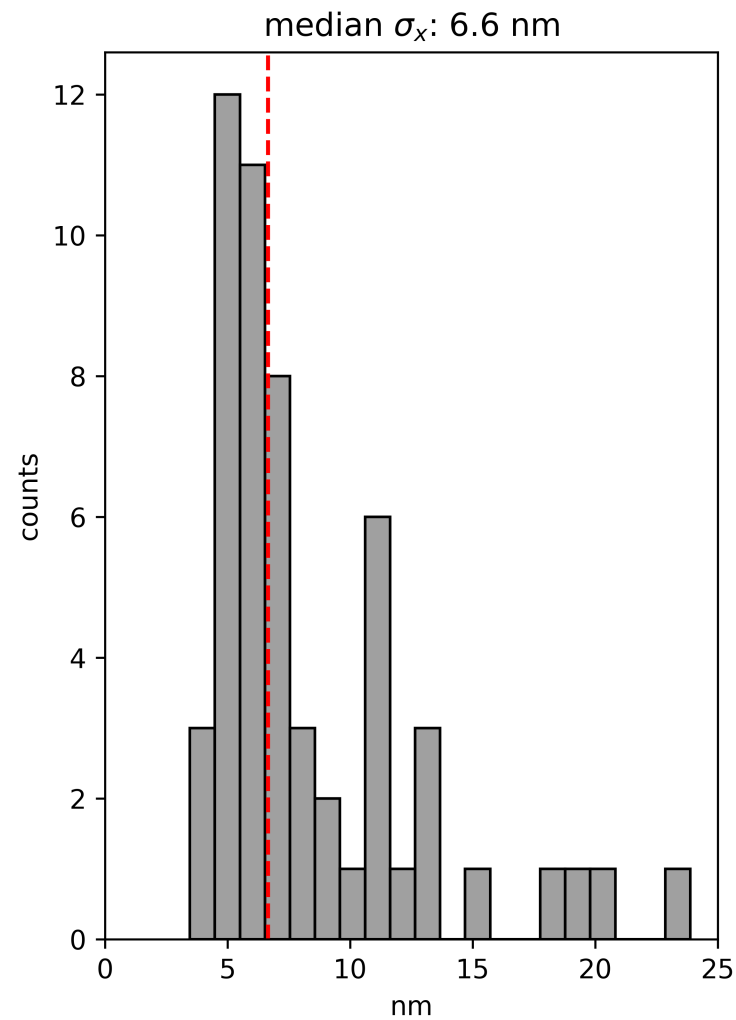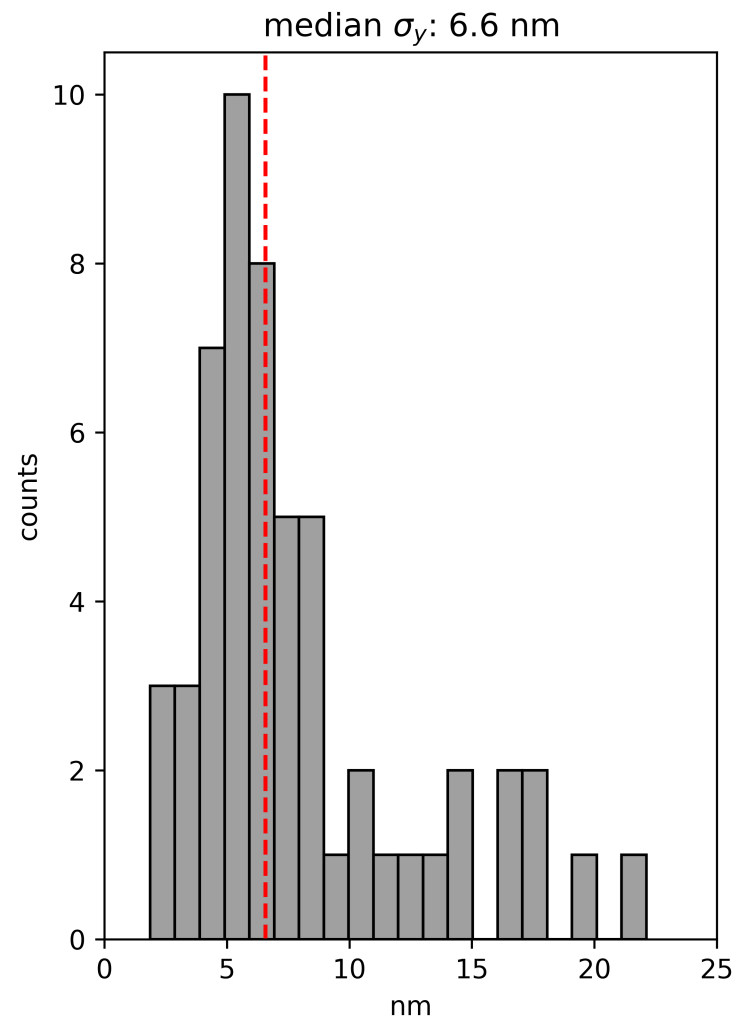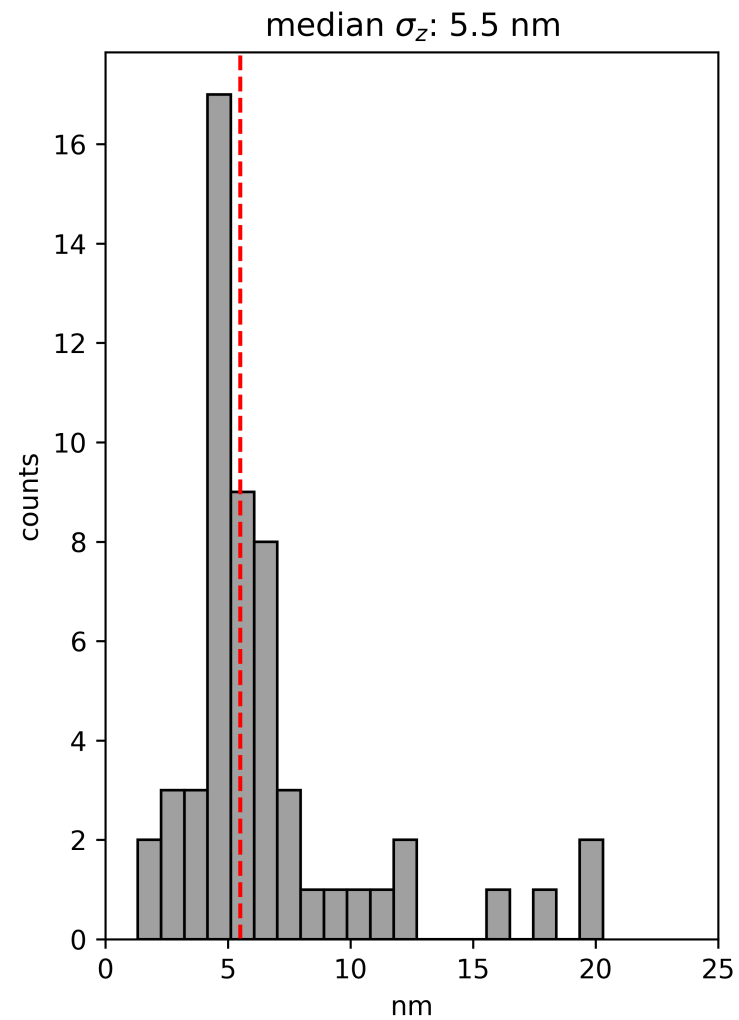

Supplement: Supplementary file 4 — Supplementary Software 1 [file 41467_2025_57729_MOESM4_ESM.zip › Scripts/TableS2_MINFLUX/231123_example_processed/_locprec_plot_summary.pdf]

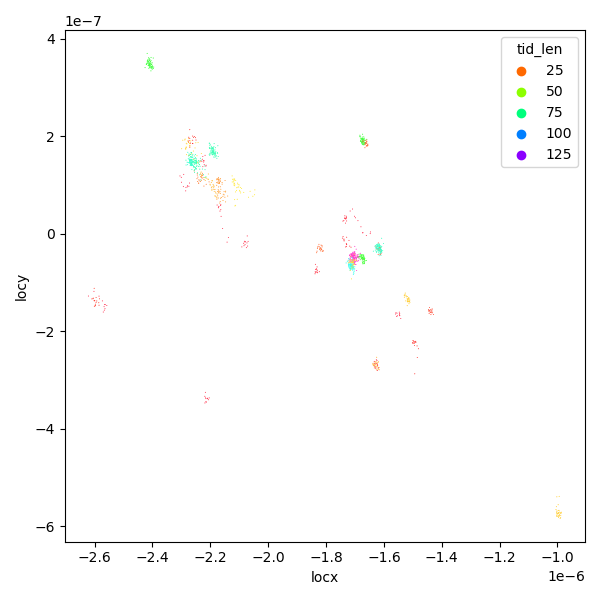

Supplement: Supplementary file 4 — Supplementary Software 1 [file 41467_2025_57729_MOESM4_ESM.zip › Scripts/TableS2_MINFLUX/231123_example_processed/231123-123107_minflux_loc_xy_plot.png]

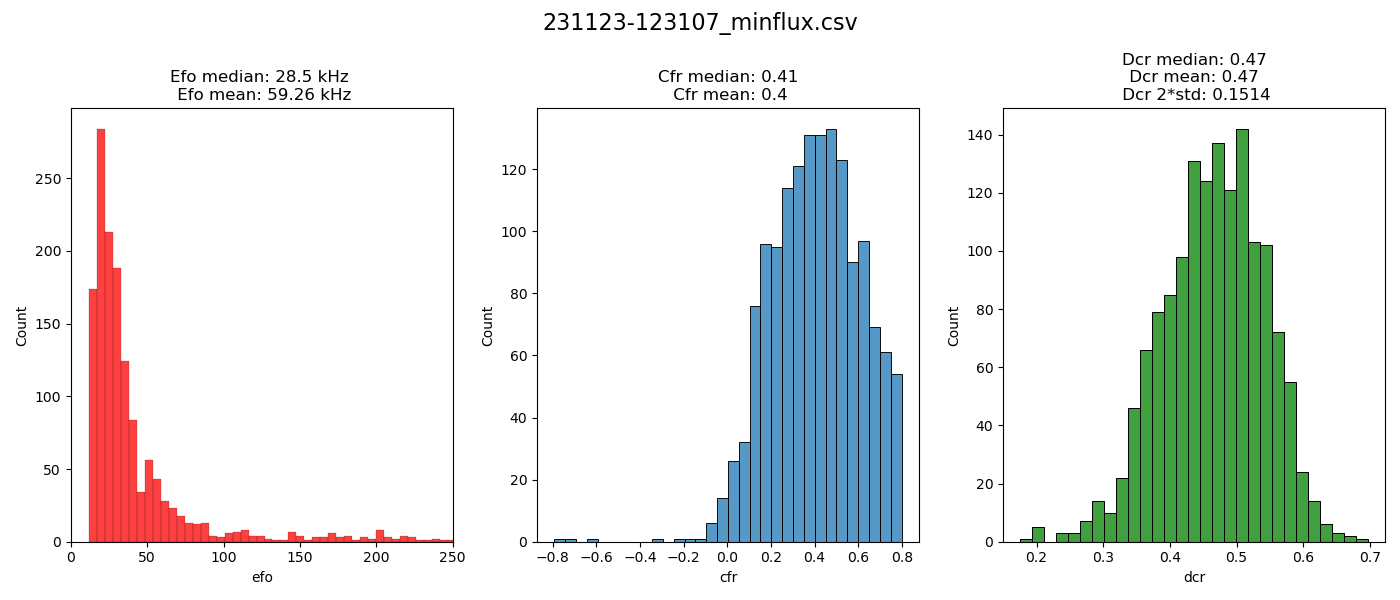

Supplement: Supplementary file 4 — Supplementary Software 1 [file 41467_2025_57729_MOESM4_ESM.zip › Scripts/TableS2_MINFLUX/231123_example_processed/231123-123107_minflux_efo_cfr_dcr.png]

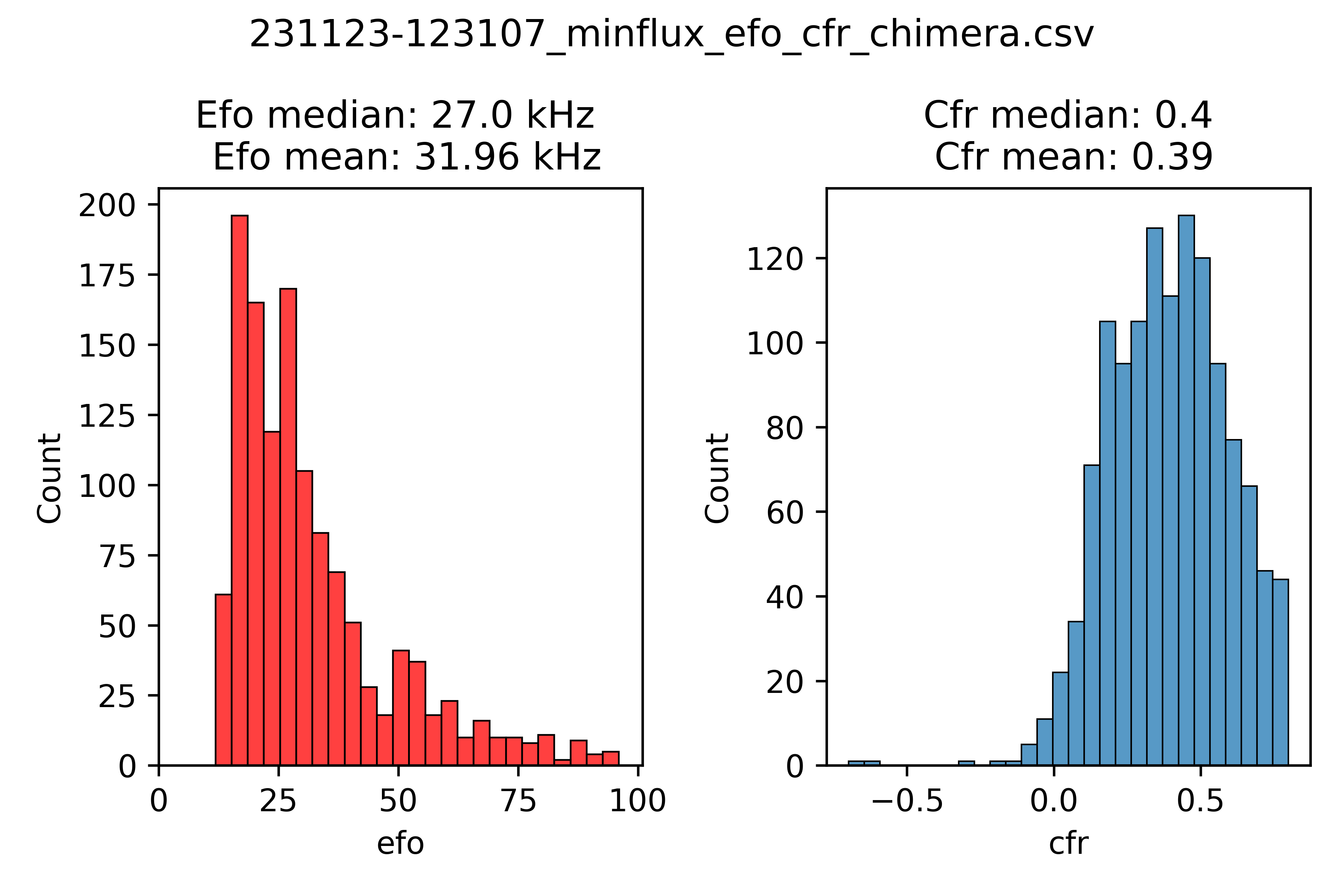

Supplement: Supplementary file 4 — Supplementary Software 1 [file 41467_2025_57729_MOESM4_ESM.zip › Scripts/TableS2_MINFLUX/231123_example_processed/231123-123107_minflux_efo_cfr_chimera.png]

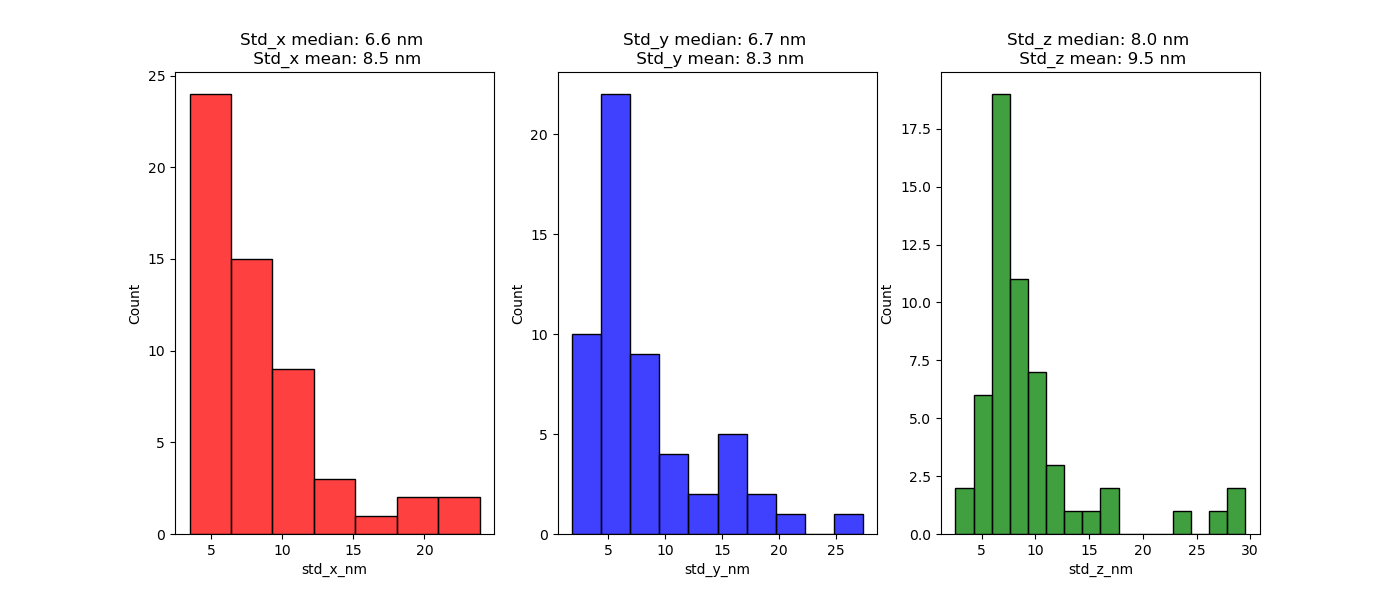

Supplement: Supplementary file 4 — Supplementary Software 1 [file 41467_2025_57729_MOESM4_ESM.zip › Scripts/TableS2_MINFLUX/231123_example_processed/231123-123107_minflux_locprec.png]

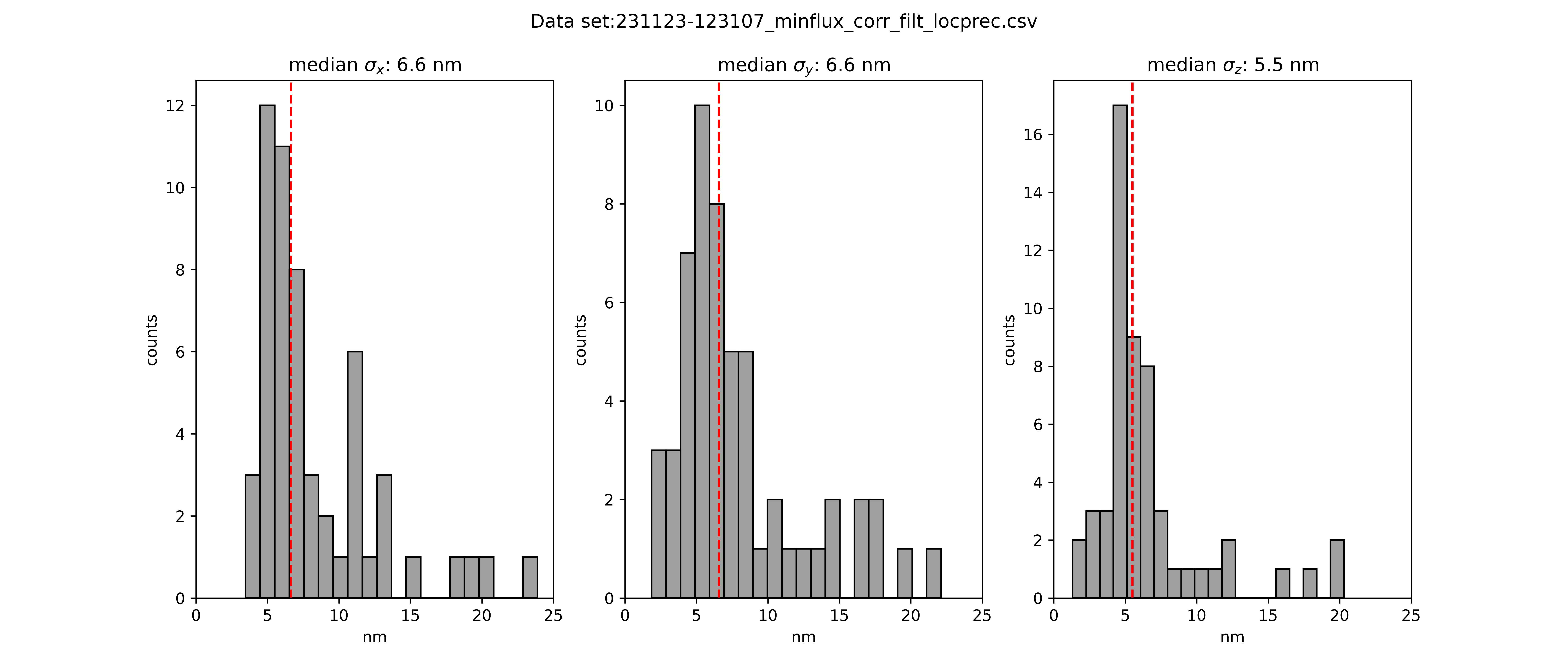

Supplement: Supplementary file 4 — Supplementary Software 1 [file 41467_2025_57729_MOESM4_ESM.zip › Scripts/TableS2_MINFLUX/231123_example_processed/231123-123107_minflux_corr_filt_locprec.png]

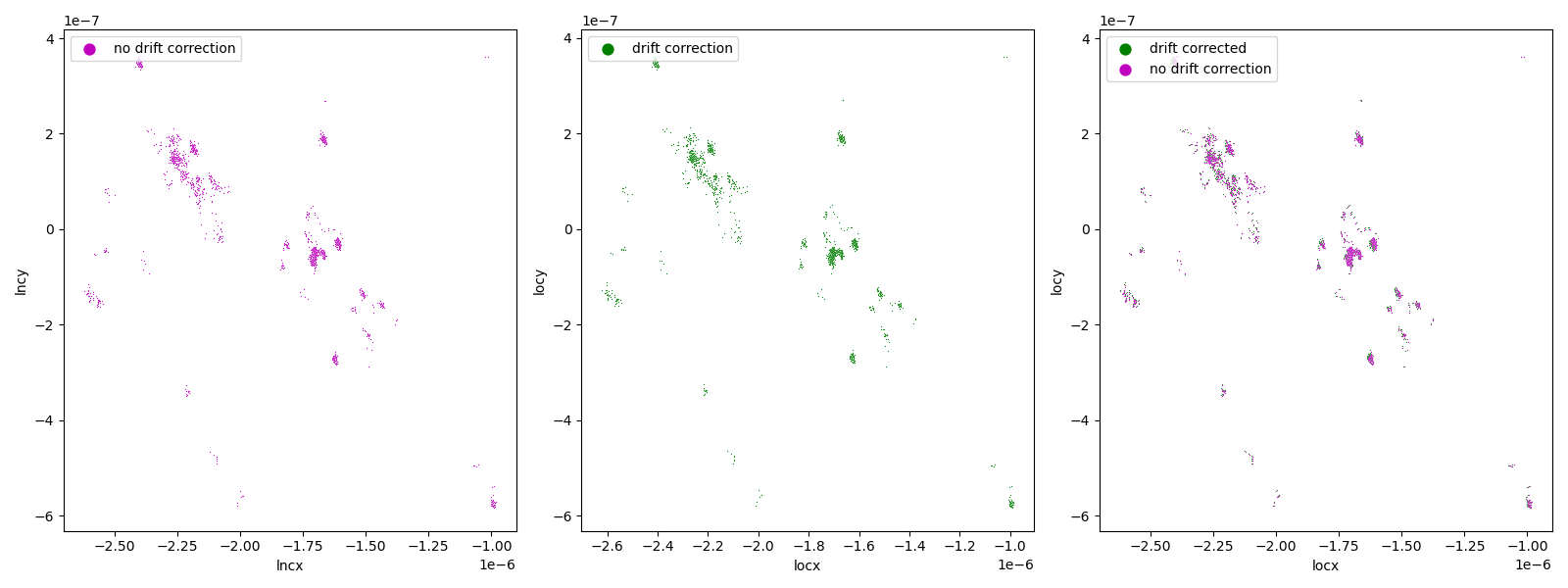

Supplement: Supplementary file 4 — Supplementary Software 1 [file 41467_2025_57729_MOESM4_ESM.zip › Scripts/TableS2_MINFLUX/231123_example_processed/231123-123107_minflux_loc_xy_plotdrift.png]

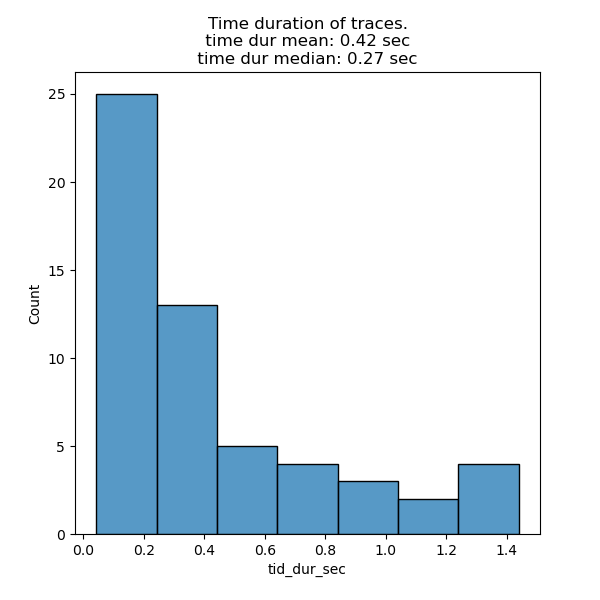

Supplement: Supplementary file 4 — Supplementary Software 1 [file 41467_2025_57729_MOESM4_ESM.zip › Scripts/TableS2_MINFLUX/231123_example_processed/231123-123107_minflux_locprec_tracedur.png]

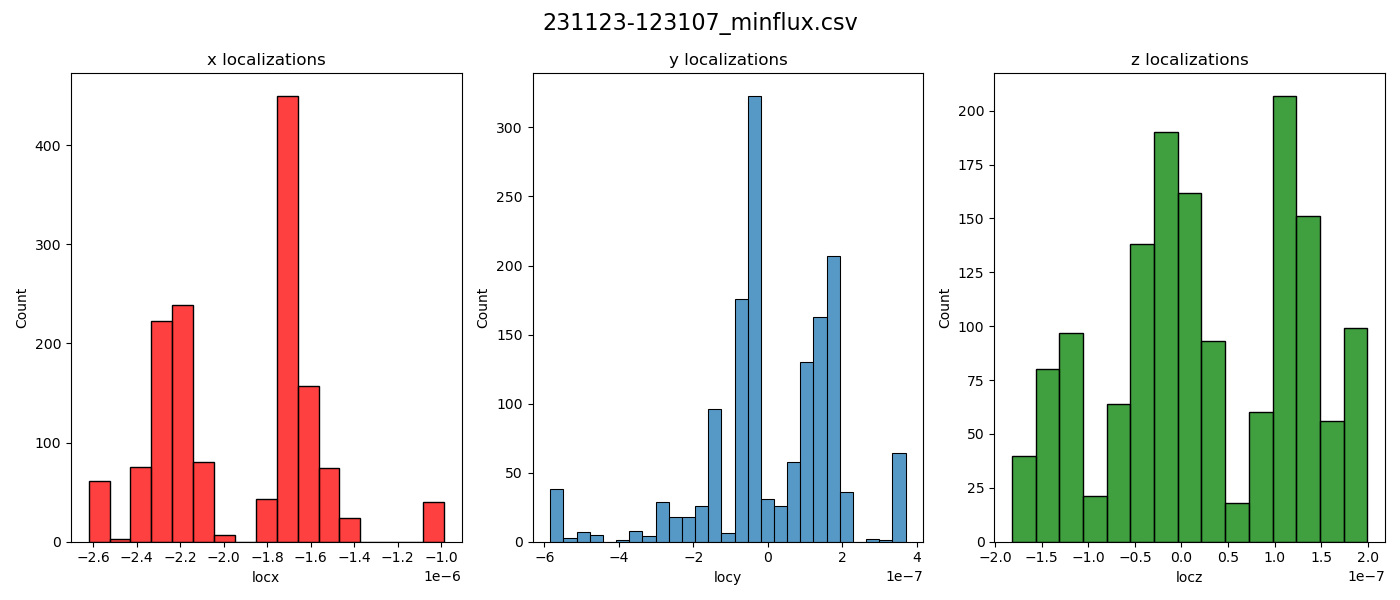

Supplement: Supplementary file 4 — Supplementary Software 1 [file 41467_2025_57729_MOESM4_ESM.zip › Scripts/TableS2_MINFLUX/231123_example_processed/231123-123107_minflux_locx_locy_locz.png]

# 231123-123107\_minflux\_efo\_cfr\_chimera.csv

Efo median: 27.0 kHz  
Efo mean: 31.96 kHz

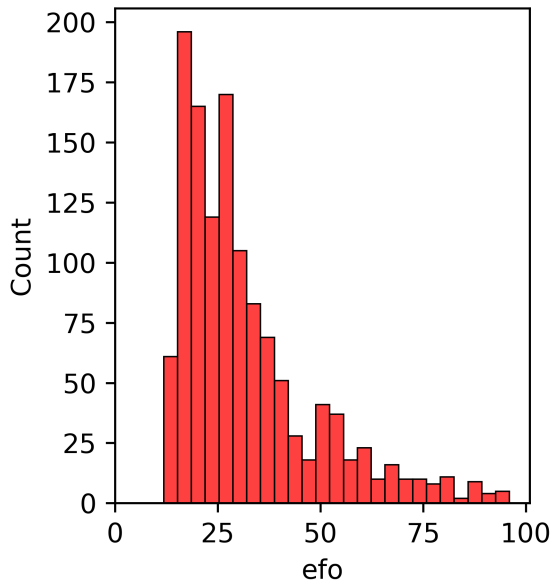

Cfr median: 0.4  
Cfr mean: 0.39

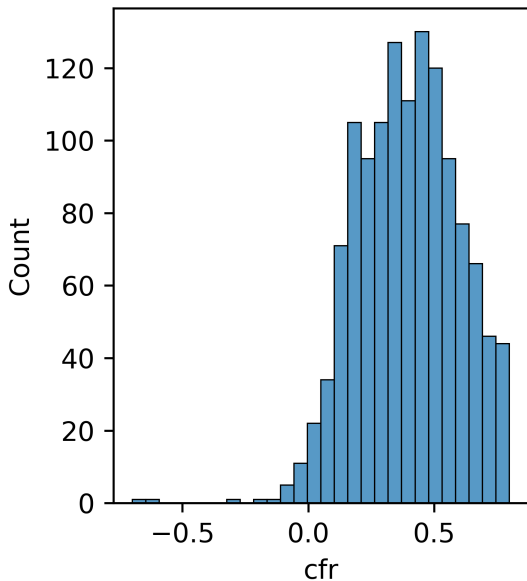

Supplement: Supplementary file 4 — Supplementary Software 1 [file 41467_2025_57729_MOESM4_ESM.zip › Scripts/TableS2_MINFLUX/231123_example_processed/231123_efo_cfr_plot_summary.pdf]
