# Supplementary material for: Structural mechanisms for centrosomal recruitment and organization of the microtubule nucleator γ-TuRC
Source: Nat Commun. 2025 Mar 12;16:2453. doi: 10.1038/s41467-025-57729-2 (PMC11903878; doi:10.1038/s41467-025-57729-2)
Supplement: Supplementary file 5 — Reporting Summary [file 41467_2025_57729_MOESM5_ESM.pdf]

nature portfolio

Corresponding author(s): Stefan Pfeffer, Elmar Schiebel

Last updated by author(s): Feb 5, 2025

Reporting Summary

Nature Portfolio wishes to improve the reproducibility of the work that we publish. This form provides structure for consistency and transparency in reporting. For further information on Nature Portfolio policies, see our Editorial Policies and the Editorial Policy Checklist.

Statistics

For all statistical analyses, confirm that the following items are present in the figure legend, table legend, main text, or Methods section.

n/a	Confirmed	
☐	☒	The exact sample size (*n*) for each experimental group/condition, given as a discrete number and unit of measurement
☐	☒	A statement on whether measurements were taken from distinct samples or whether the same sample was measured repeatedly
☐	☒	The statistical test(s) used AND whether they are one- or two-sided<br>*Only common tests should be described solely by name; describe more complex techniques in the Methods section.*
☒	☐	A description of all covariates tested
☒	☐	A description of any assumptions or corrections, such as tests of normality and adjustment for multiple comparisons
☐	☒	A full description of the statistical parameters including central tendency (e.g. means) or other basic estimates (e.g. regression coefficient) AND variation (e.g. standard deviation) or associated estimates of uncertainty (e.g. confidence intervals)
☐	☒	For null hypothesis testing, the test statistic (e.g. *F*, *t*, *r*) with confidence intervals, effect sizes, degrees of freedom and *P* value noted<br>*Give P values as exact values whenever suitable.*
☒	☐	For Bayesian analysis, information on the choice of priors and Markov chain Monte Carlo settings
☒	☐	For hierarchical and complex designs, identification of the appropriate level for tests and full reporting of outcomes
☒	☐	Estimates of effect sizes (e.g. Cohen's *d*, Pearson's *r*), indicating how they were calculated

Our web collection on statistics for biologists contains articles on many of the points above.

Software and code

Policy information about availability of computer code

Data collection	Cryo-EM data was collected using EPU (version 2.6, SPA) or SerialEM 3.8 for cellular cryo-ET data and EPU Tomography (version 5.5.0.1941) for cryo-ET data of purified centrosomes, on a Krios TEM equipped with a Gatan K3 camera operated by Gatan Microscopy Suite (version 3.32). Immunofluorescent images were acquired using Software WoRx (6.1). Data for immunoblots was collected using LAS4000 (2.1). Images of Expansion microscope were collected by Leica TCS SP8 STED microscpe. MINFLUX measurements were performed on an Abberior MINFLUX microscope (Abberior Instruments and controlled by the software Imspector (v16.3.15636-m2205-win64-MINFLUX). Chromatography runs were performed using an ÄktaPure instrument (Cytiva). Mass photometry was measured using a Refeyn TwoMP mass photometer (Refeyn Ltd, Oxford, UK). Images of STED and confocal images were collected by Leica TCS SP8 STED microscope. Data for fluorescence recovery after photobleaching (FRAP) were collected by Zeiss LSM 780 microscope. Mass spectrometry (MS) data were collected in Xcalibur 4.7.69.37
Data analysis	EM data were processed using Warp & M 1.09 Relion 3.0 and 3.1, MotionCor2 v1.0.5, Gctf 1.06 and spIsoNet v1.0. Selected maps were visualized using OccuPy v0.1.13. Tilt-series alignment was carried out in IMOD 4.11 & template matching and reference free-alignment in PyTOM v0.971. Spatial analysis of tomographic data was performed in Matlab R2023a. All atomic model and density-related analysis and visualization was done using UCSF Chimera 1.14 and UCSF ChimeraX 1.3 & 1.7.1. Model building was performed using Coot 0.9.2 and Namdinator. AlphaFold predictions were carried out using AlphaFold Multimer 2.3.1 and 2.3.2 as well as AlphaFold3 release 2024.08.19. Curve visualisation was done using R 4.1.2 in RStudio 2022.02.0 and Prism 9.1. immunofluorescent data was analyzed using Fiji software and deconvoluted by Huygens' Deconvolution software (SVI Inc.). The Äkta pure system was controlled by Unicorn software (version 7.9). Mass photometry data were analysed using Refeyn DiscoverMP 2024 R1 software (Refeyn Ltd, Oxford, UK). MINFLUX data visualization was performed by using a pipeline of pyMINFLUX version 0.3.0 and the visualization software Paraview 5.11.2. Central axis fitting for MINFLUX signal was performed in UCSF Chimera 1.14. MS data were processed using MSConvert, MSFragger (v4.0), FragPipe (v21.1), philosopher (v5.1.0) and IonQuant (v1.10.12).

Custom scripts used in this study are available with the article.

For manuscripts utilizing custom algorithms or software that are central to the research but not yet described in published literature, software must be made available to editors and reviewers. We strongly encourage code deposition in a community repository (e.g. GitHub). See the Nature Portfolio guidelines for submitting code & software for further information.

Data

Policy information about availability of data

All manuscripts must include a data availability statement. This statement should provide the following information, where applicable:

- Accession codes, unique identifiers, or web links for publicly available datasets
- A description of any restrictions on data availability
- For clinical datasets or third party data, please ensure that the statement adheres to our policy

The atomic coordinates and the cryo-EM densities for the X. laevis γ-TuRC with stoichiometric NEDD1/N-GCP3/MZT1 grapnel generated in this study have been deposited in the Protein Data Bank and the Electron Microscopy Data Bank under accession codes PDB: 9I8N [https://www.rcsb.org/structure/9I8N] and EMD-52730 [https://www.ebi.ac.uk/emdb/EMD-52730] (consensus reconstruction), PDB: 9I8M [https://www.rcsb.org/structure/9I8M] and EMD-52729 [https://www.ebi.ac.uk/emdb/EMD-52729] (focused refinement on grapnel with spoke 9-12 GRIP1), and EMD-52728 [https://www.ebi.ac.uk/emdb/EMD-52728] (focused refinement on spoke 12-14). The cryo-EM densities and associated models (if applicable) derived from cryo-ET data generated in this study have been deposited in the Electron Microscopy Data Bank and Protein Data Bank under accession codes EMD-52722 [https://www.ebi.ac.uk/emdb/EMD-52722] (γ-TuRCs from cells), EMD-52717 [https://www.ebi.ac.uk/emdb/EMD-52717] (KE37 γ-TuRC consensus refinement), PDB: 9I8G [https://www.rcsb.org/structure/9I8G] and EMD-52718 [https://www.ebi.ac.uk/emdb/EMD-52718] (KE37 γ-TuRC inwards conformation), PDB: 9I8H [https://www.rcsb.org/structure/9I8H] and EMD-52719 [https://www.ebi.ac.uk/emdb/EMD-52719] (KE37 γ-TuRC outwards conformation), EMD-52721 [https://www.ebi.ac.uk/emdb/EMD-52721] (RPE1 wild-type γ-TuRC), EMD-52720 [https://www.ebi.ac.uk/emdb/EMD-52720] (RPE1 CDK5RAP2-/- γ-TuRC), and EMD-52723 [https://www.ebi.ac.uk/emdb/EMD-52723] (MTTs from cells; all particles as well as topological classes). The models predicted by AlphaFold and AlphaFold-Multimer generated in this study have been deposited in the ModelArchive database (https://modelarchive.org/) with the identifiers ma-odixd [https://modelarchive.org/doi/10.5452/ma-odixd] (human NEDD1C/N-GCP3/MZT1 grapnel), ma-imf31 [https://modelarchive.org/doi/10.5452/ma-imf31] (X. laevis NEDD1C/N-GCP3/MZT1 grapnel), ma-phn3m [https://modelarchive.org/doi/10.5452/ma-phn3m] (p1 N-GCP3/MZT1 module with GCP4, GCP5 and GCP6 GRIP1), ma-rmctb [https://modelarchive.org/doi/10.5452/ma-rmctb] (p2 N-GCP3/MZT1 module with GCP4, GCP5 and GCP6 GRIP1), ma-1vc4y [https://modelarchive.org/doi/10.5452/ma-1vc4y] (GCP2 and GCP3 GRIP1 with N-GCP6), ma-89x6h [https://modelarchive.org/doi/10.5452/ma-89x6h] (NEDD1C tetramer with N-GCP3/MZT1 module and Augmin TIII), ma-rwmkh [https://modelarchive.org/doi/10.5452/ma-rwmkh] (POC5 dimer with centrin 2 and Augmin TII N-clamp), ma-2zl3w [https://modelarchive.org/doi/10.5452/ma-2zl3w] (γ-TuSC with MZT2 and CDK5RAP2 dimer), ma-8bwwq [https://modelarchive.org/doi/10.5452/ma-8bwwq] (N-GCP2/MZT2 module), ma-yzz9w [https://modelarchive.org/doi/10.5452/ma-yzz9w] (N-GCP5/MZT1 module), ma-14965 [https://modelarchive.org/doi/10.5452/ma- 14965] (N-GCP3/MZT1 module) and ma-z68wf [https://modelarchive.org/doi/10.5452/ma-z68wf] (X. laevis GCP6). Mass spectrometry data of purified X. laevis γ-TuRC are available as Supplementary Dataset 1 and have been deposited to the ProteomeXchange Consortium via the PRIDE 99 partner repository with the dataset identifier PXD060119 [https://www.ebi.ac.uk/pride/archive/projects/PXD060119]. Tilt series of centrosomes in vitreous sections of HeLa cells used in this study are available in the EMPIARC database under accession code 200003 [http://www.emdb-china.org.cn/dataEmpiarc?code=EMPIARC-200003]. Source data are provided with this paper.

Research involving human participants, their data, or biological material

Policy information about studies with human participants or human data. See also policy information about sex, gender (identity/presentation), and sexual orientation and race, ethnicity and racism.

Reporting on sex and gender	*Use the terms sex (biological attribute) and gender (shaped by social and cultural circumstances) carefully in order to avoid confusing both terms. Indicate if findings apply to only one sex or gender; describe whether sex and gender were considered in study design; whether sex and/or gender was determined based on self-reporting or assigned and methods used. Provide in the source data disaggregated sex and gender data, where this information has been collected, and if consent has been obtained for sharing of individual-level data; provide overall numbers in this Reporting Summary. Please state if this information has not been collected. Report sex- and gender-based analyses where performed, justify reasons for lack of sex- and gender-based analysis.*
Reporting on race, ethnicity, or other socially relevant groupings	*Please specify the socially constructed or socially relevant categorization variable(s) used in your manuscript and explain why they were used. Please note that such variables should not be used as proxies for other socially constructed/relevant variables (for example, race or ethnicity should not be used as a proxy for socioeconomic status). Provide clear definitions of the relevant terms used, how they were provided (by the participants/respondents, the researchers, or third parties), and the method(s) used to classify people into the different categories (e.g. self-report, census or administrative data, social media data, etc.) Please provide details about how you controlled for confounding variables in your analyses.*
Population characteristics	*Describe the covariate-relevant population characteristics of the human research participants (e.g. age, genotypic information, past and current diagnosis and treatment categories). If you filled out the behavioural & social sciences study design questions and have nothing to add here, write "See above."*
Recruitment	*Describe how participants were recruited. Outline any potential self-selection bias or other biases that may be present and how these are likely to impact results.*
Ethics oversight	*Identify the organization(s) that approved the study protocol.*

Note that full information on the approval of the study protocol must also be provided in the manuscript.

Field-specific reporting

Please select the one below that is the best fit for your research. If you are not sure, read the appropriate sections before making your selection.

☒ Life sciences ☐ Behavioural & social sciences ☐ Ecological, evolutionary & environmental sciences

For a reference copy of the document with all sections, see nature.com/documents/nr-reporting-summary-flat.pdf

Life sciences study design

All studies must disclose on these points even when the disclosure is negative.

Sample size	For EM data, no statistical method was chosen to determine the sample size. The number of micrographs or tilt-series was chosen to obtain a number of particles sufficient to reconstruct a 3D density of the gamma-TuRC at the stated resolution and to obtain an ab-initio model of the NEDD1 grapnel in case of negative stain EM data. Collected cryo-EM SPA data contained 29516 images of gamma-TuRC with NEDD1, corresponding to one four-day microscopy sessions with parameters specified in the method section.<br>The initial number of picked particles was 11206035. After multiple rounds of 3D classification, we reached a final dataset of 299022 particles that were used for refinement in RELION. The final reconstructions reached resolution that was estimated by the Gold Standard FSC method. AlphaFold predictions produced 5x5 (AlphaFold2) or 5 (AlphaFold3) models per construct. For immunofluorescent experiment, images were acquired in number that is sufficient for statistical analysis (as specified in figure legend and Methods).<br>Collected raw datasets contained 62 KE37 tilt-series, 20 RPE wild-type tilt-series and 21 RPE1 CDK5RAP2-/- tilt-series. Collected raw datasets of cellular tomography data from HCT116 cells contained 13 tilt-series and raw datasets of cellular tomography data from HeLa cells (EMPIARC-200003) consisted of 46 tilt-series. Datasets have been aquired and processed as described in the method section. For MINFLUX, raw data contained datasets of 35 positions aquired over 4 experiments.<br>For negative stain EM NEDD1 grapnel processing, the initial number of picked particles after autopicking was 37873. Following 2 iterative rounds of 2D classifications a final selection of 464 particles was used for generating a reference free ab-initio model.<br>No statistical method was used to determine the sample size for MS analysis. The sample size was sufficient to detect proteins at stoichiometry considerably lower than established γ-TuRC components during MS analysis.
Data exclusions	For cryo-EM SPA and negative stain EM, all EM images were used for particle picking. Cryo-EM particle selection was performed in several 3D classification rounds, and the quality of resulting 3D maps was the exclusion criterion, as is standard image processing practice in cryo-EM. Negative stain EM particle selection was performed in consecutive 2D classification rounds and the exclusion criterion was based on the shape of the classaverages. For cryo-electron tomography analysis, tomograms were excluded based on alignment quality and performance in template matching attempts. Subtomogram selection after template matching was performed through 3D classification and data quality of the resulting 3D maps has been used as exclusion criterion. Data exclusions of MINFLUX data for distance analysis was based on whether datasets could be drift corrected and on the structural arrangement of MINFLUX data, as well as the ability to fit a central axis for individual centrosomes. No data was excluded from MS analysis.
Replication	Cryo-EM SPA and negative stain EM data were acquired on one grid. 3D densities are averages of hundreds of thousands of particles and thus repetitions of the experiments were not necessary. Expansion microscopy experiments were repeated at least twice. Immunofluorescence and immunoblot experiments were repeated at least three times (unless specified in figure legend and Methods). Cryo-EM tomography data were aquired over three sessions for KE37 tilt-series, one session for RPE wild-type and RPE1 CDK5RAP2-/-, respectively, and two sessions for cellular tomography data of HCT116 cells
Randomization	Positions for image acquisition in cryo-EM and negative stain EM were selected based on the presence of the particles. MINFLUX data were aquired randomly. All immunofluorescence images were acquired randomly. Randomization was not applicable to experiments not comparing different samples.
Blinding	EM and MS analysis was not blinded because it was performed computationally

Reporting for specific materials, systems and methods

We require information from authors about some types of materials, experimental systems and methods used in many studies. Here, indicate whether each material, system or method listed is relevant to your study. If you are not sure if a list item applies to your research, read the appropriate section before selecting a response.

Materials & experimental systems

n/a	Involved in the study
☐	☒ Antibodies
☐	☒ Eukaryotic cell lines
☒	☐ Palaeontology and archaeology
☐	☒ Animals and other organisms
☒	☐ Clinical data
☒	☐ Dual use research of concern
☒	☐ Plants

Methods

n/a	Involved in the study
☒	☐ ChIP-seq
☒	☐ Flow cytometry
☒	☐ MRI-based neuroimaging

Antibodies

Antibodies used

Primary antibodies used in this study were: Mouse α-tubulin (Proteintech 660311-1-Ig; ExM: 1:500), rabbit α-tubulin (Proteintech 11224-1-AP; ExM: 1:500), mouse
γ-tubulin (abcam Ab27074; IF: 1:500; ExM: 1:500; WB: 1:1000), rat HA (Merck 11867423001; IF: 1:1000; ExM: 1:1000; WB: 1:1000), rabbit POC5 (Bethyl A303-341A-T;
IF 1:2000; ExM: 1:2000), rabbit HAUS4 (Proteintech 20104-1-AP; IF: 1:500; ExM: 1:500; WB: 1:1000), mouse ACTIN (WB 1:1000), rabbit CDK5RAP2 (Merck 2952319;
IF: 1:500; ExM: 1:500), mouse NEDD1(Santa Cruz sc-100961; IF: 1:500; ExM: 1:500), mouse GCP4 (Santa Cruz sc-271876; ExM: 1:500), mouse HIS (QIAGEN
169033106; WB 1:1000), Rabbit GAPDH (Cell Signaling Technology 14C10; WB: 1:1000), Rabbit GCP3 (Proteintech 15719-1-AP; WB: 1:1000), Rabbit HAUS1
(ATLAS ANTIBODIES HPA040601; WB: 1:1000), mouse GCP5 (Invitrogen PA5-83495; WB: 1:1000), Rabbit Aurora A (Cell Signaling Technology 1G4; WB: 1:1000),
Rabbit CDK1 (Proteintech 10762-1-AP; WB: 1:1000), Rabbit pH3 (Cell Signaling Technology D2C8; WB: 1:1000), Mouse ki67 (Santa Cruz Biotechnology sc-23900; WB: 1:1000),
Rabbit STREP (Abcam EPR28119-43; WB: 1:1000; IF: 1:500; ExM 1:200), . Secondary antibodies used in this study were: Mouse Alexa Fluor Plus 488 (Thermo Fisher Scientific A-11001; 1:500),
rabbit Alexa Flour Plus 488 (Thermo Fisher Scientific A-11008; 1:500), mouse Alexa Flour Plus 555 (Thermo Fisher Scientific A32727; 1:500), rabbit Alexa Flour Plus 555 (Thermo Fisher
Scientific A32732; 1:500), mouse Alexa Flour Plus 647 (Thermo Fisher Scientific A32728; 1:500), rabbit Alexa Flour Plus 647 (Thermo Fisher Scientific A32733; 1:500),
mouse Abberior STAR 635P (Abberior ST635P-1001-500UG; 1:500), rabbit Abberior STAR 635P (Abberior ST635P-1002-500UG; 1:500), mouse True blot (Rockland 18-8817-33; WB: 1:1000). Anti-ALFA HRP conjugated anti-alpaca (Nanotag N1505HRP, 1:5000).
Secondary HRP-conjugated antibodies used in this study for immunoblotting are HRP anti-mouse (Jackson 715-035-151; 1:5000), HRP anti-rabbit (Jackson 711-035-152; 1:5000) and HRP anti-rat (Jackson 712-035-153; 1:5000).

Validation

Homemade rabbit γ-tubulin antibody was validated previously in the publication: doi: 10.1038/s41586-019-1896-6
All commercial antibodies were validated by the manufacturers:
Mouse α-tubulin (clone 1E4C11):
https://www.ptgcn.com/products/tubulin-Alpha-Antibody-66031-1-Ig.htm
rabbit α-tubulin:
https://www.ptgcn.com/products/TUBA1B-Antibody-11224-1-AP.htm
mouse γ-tubulin (TU-30):
https://www.abcam.com/en-de/products/primary-antibodies/gamma-tubulin-antibody-tu-30-ab27074
rabbit POC5:
https://www.thermofisher.com/antibody/product/POC5-Antibody-Polyclonal/A303-341A
rabbit HAUS4:
https://www.ptglab.com/de/products/HAUS4-Antibody-20104-1-AP.htm
rabbit CDK5RAP2 (06-1398):
https://www.merckmillipore.com/DE/de/product/Anti-CDK5RAP2-Antibody,MM_NF-06-1398
mouse NEDD1 (39-J):
https://www.scbt.com/zh/p/nedd1-antibody-39-j
mouse GCP4 (D-5):
https://www.scbt.com/zh/p/gcp4-antibody-d-5
mouse ACTIN (clone AC-74):
https://www.sigmaaldrich.com/DE/en/product/sigma/a2228
rat HA:
https://www.sigmaaldrich.com/DE/en/product/roche/roahaha
mouse HIS:
https://www.qiagen.com/us/products/discovery-and-translational-research/protein-purification/tagged-protein-expression-purification-detection/anti-his-antibodies-bsa-free?catno=34660
Mouse Alexa Fluor Plus 488:
https://www.thermofisher.com/antibody/product/Goat-anti-Mouse-IgG-H-L-Cross-Adsorbed-Secondary-Antibody-Polyclonal/A-11001
rabbit Alexa Flour Plus 488:
https://www.thermofisher.com/antibody/product/Goat-anti-Rabbit-IgG-H-L-Cross-Adsorbed-Secondary-Antibody-Polyclonal/A-11008
mouse Alexa Flour Plus 555:
https://www.thermofisher.com/antibody/product/Goat-anti-Mouse-IgG-H-L-Highly-Cross-Adsorbed-Secondary-Antibody-Polyclonal/A32727
rabbit Alexa Flour Plus 555:
https://www.thermofisher.com/antibody/product/Goat-anti-Rabbit-IgG-H-L-Highly-Cross-Adsorbed-Secondary-Antibody-Polyclonal/A32732
mouse Alexa Flour Plus 647:
https://www.thermofisher.com/antibody/product/Goat-anti-Mouse-IgG-H-L-Highly-Cross-Adsorbed-Secondary-Antibody-Polyclonal/A32728
rabbit Alexa Flour Plus 647:
https://www.thermofisher.com/antibody/product/Goat-anti-Rabbit-IgG-H-L-Highly-Cross-Adsorbed-Secondary-Antibody-Polyclonal/A32733
mouse Abberior STAR 635P:
https://abberior.shop/abberior-STAR-635P-goat-anti-mouse-IgG-500-ll-1-mg-ml
rabbit Abberior STAR 635P:

https://abberior.shop/abberior-STAR-635P-goat-anti-rabbit-IgG-500-ll-1-mg-ml
HRP anti-mouse:
https://www.jacksonimmuno.com/catalog/products/715-035-151
HRP anti-rabbit:
https://www.jacksonimmuno.com/catalog/products/711-035-152
HRP anti-rat:
https://www.jacksonimmuno.com/catalog/products/712-035-153
Rabbit GAPDH:
https://www.cellsignal.com/products/primary-antibodies/gapdh-14c10-rabbit-mab/2118
Rabbit GCP3:
Need to search for the official website (Proteintech 15719-1-AP)
Rabbit HAUS1:
https://www.atlasantibodies.com/products/primary-antibodies/triple-a-polyclonals/anti-haus1-antibody-hpa040601/
mouse GCP5:
Need to search for the official website (Invitrogen PA5-83495)
Rabbit Aurora A:
https://www.cellsignal.com/products/primary-antibodies/aurora-a-aik-1g4-rabbit-mab/4718
Rabbit CDK1:
Need to search for the official website (Proteintech 10762-1-AP)
Rabbit pH3:
https://www.cellsignal.com/products/primary-antibodies/phospho-histone-h3-ser10-d2c8-xp-rabbit-mab/3377
Mouse ki67:
https://www.scbt.com/p/ki67-antibody-ki-67
ALFA-HRP:
https://www.sysy.com/product/N1505-HRP
Rabbit STREP:
https://www.abcam.com/en-us/products/primary-antibodies/strep-tag-ii-antibody-epr28119-43-ab307676
mouse True blot:
https://www.rockland.com/categories/trueblot/mouse-trueblot-ultra-anti-mouse-ig-hrp-18-8817-30/

Eukaryotic cell lines

Policy information about cell lines and Sex and Gender in Research

Cell line source(s)

SF21 insect cell line was obtained from the EMBL protein expression facility. Sf9 insect cells (Spodoptera frugiperda) Sigma-Aldrich. RPE1 cell line, HEK293T cell line and HEK GP2-293 cell line are as described (https://doi.org/10.1038/s41467-020-14767-2). KE37 cell line is as described (https://doi.org/10.1002/j.1460-2075.1986.tb04533.x). HCT116 cell line is as described (https://doi.org/10.15252/embj.2021109738).

Authentication

We verified the cell lines according to the morphology by light microscopy.

Mycoplasma contamination

All cell lines were negative in the mycoplasma contamination test.

Commonly misidentified lines
(See ICLAC register)

No commonly misidentified lines were used in this study.

Animals and other research organisms

Policy information about studies involving animals; ARRIVE guidelines recommended for reporting animal research, and Sex and Gender in Research

Laboratory animals

Xenopus laevis (Xenopus1, Dexter, MI, USA), female, sexually mature, i.e. more than 9 months old.

Wild animals

No wild animals were used in this study.

Reporting on sex

Female Xenopus laevis were used to produce eggs.

Field-collected samples

No field-collected samples were used in this study.

Ethics oversight

Husbandry of Xenopus laevis female frogswas approved by the the City of Bonn, Germany, quoting §11 Abs. 1, Nr.1 of the German law for animal protection under file reference 76-5/2022/, Amt für Umwelt und Stadtgrün according to EU guideline 2010/63 for aquatic anura. Hormone applications for egg production were approved by the Landesamt für Natur, Umwelt und Verbraucherschutz Nordrhein-Westfalen, Germany, under the file reference 81-02.04.40.2022.VG027

Note that full information on the approval of the study protocol must also be provided in the manuscript.

Plants

Seed stocks

Report on the source of all seed stocks or other plant material used. If applicable, state the seed stock centre and catalogue number. If plant specimens were collected from the field, describe the collection location, date and sampling procedures.

Novel plant genotypes

Describe the methods by which all novel plant genotypes were produced. This includes those generated by transgenic approaches, gene editing, chemical/radiation-based mutagenesis and hybridization. For transgenic lines, describe the transformation method, the number of independent lines analyzed and the generation upon which experiments were performed. For gene-edited lines, describe the editor used, the endogenous sequence targeted for editing, the targeting guide RNA sequence (if applicable) and how the editor was applied.

Authentication

Describe any authentication procedures for each seed stock used or novel genotype generated. Describe any experiments used to assess the effect of a mutation and, where applicable, how potential secondary effects (e.g. second site T-DNA insertions, mosiacism, off-target gene editing) were examined.
